# Reductive Head-to-Head Coupling of Phenylacetylenes in the Coordination Sphere of an Osmium-Polyhydride

**DOI:** 10.1021/acs.organomet.4c00480

**Published:** 2025-01-06

**Authors:** Sheila G. Curto, Miguel A. Esteruelas, Katarzyna A. Mituła-Chmielowiec, Enrique Oñate

**Affiliations:** Departamento de Química Inorgánica, Instituto de Síntesis Química y Catálisis Homogénea (ISQCH), Centro de Innovación en Química Avanzada (ORFEO−CINQA), Universidad de Zaragoza-CSIC, Zaragoza 50009, Spain

## Abstract

Complex OsH_6_(P^i^Pr_3_)_2_ releases H_2_ at 50 °C. The resulting tetrahydride OsH_4_(P^i^Pr_3_)_2_ promotes head-to-head reductive dimerization of phenylacetylenes to give the 1,4-dibranched-butenediyl derivatives OsH_2_{η^4^-[C_4_H_4_R_2_]}­(P^i^Pr_3_)_2_ (*R* = C_6_H_5_, C_6_H_4_–CF_3_, C_6_H_4_–NMe_2_). DFT calculations suggest that the formation of these compounds proceeds via five-coordinate unsaturated bis­(alkenyl)-osmium­(II)-(Kubas-type dihydrogen) intermediates, which evolve by alkenyl coupling and H–H cleavage of the dihydrogen. The reactions are sensitive to temperature and the amount of alkyne used. At higher temperatures and excess alkyne, the reductive coupling is accompanied by two dehydrogenation reactions, one at the metal center and the other involving an isopropyl substituent of a phosphine. As a result, mixtures of the dihydrides and Os­{η^4^-[C_4_H_4_R_2_]}­(P^i^Pr_3_)­{η^2^-*C*,*C*;κ^1^-*P*-[(CH_2_=CMe)­P^i^Pr_2_]}­(P^i^Pr_3_) (*R* = H, CF_3_, NMe_2_) are formed. Both families react with H_2_ to regenerate OsH_6_(P^i^Pr_3_)_2_ and release the corresponding 1,4-diarylbutane. According to these reactions, 1,4-diarylbutanes have been obtained in approximately 20% yield, by stirring phenylacetylenes with 5 mol % of OsH_6_(P^i^Pr_3_)_2_, in toluene, under 1 atm of H_2_.

## Graphical abstract

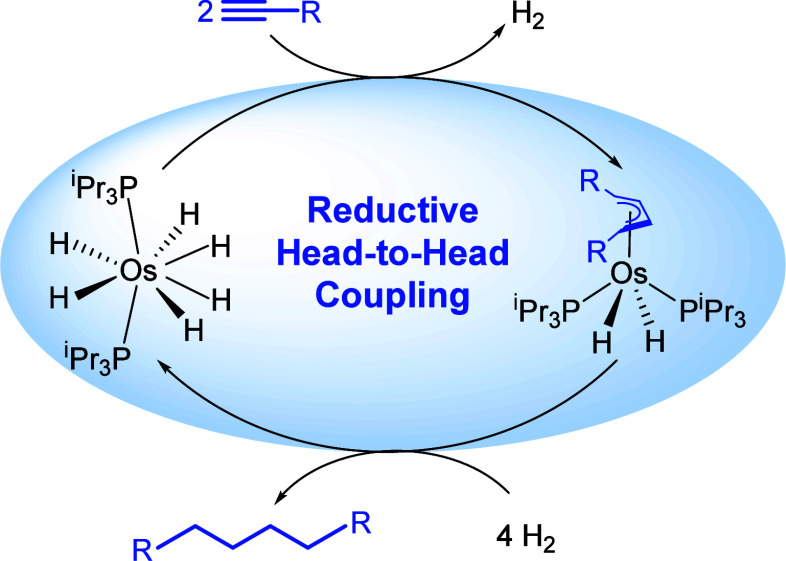

## Introduction

Terminal alkynes are easy-to-handle building blocks in organic synthesis. They increase in importance when participating in selective chemical transformations involving atom economy.^1^ Among them, direct homodimerization is gaining relevance due to the practical application of the resulting products. Three types of dimerization are known: oxidative to 1,3-diynes,^2^ redox-neutral to 1,3-enynes or 1,2,3-butatrienes,^3^ and reductive to 1,3-dienes.^4^ The latter is the most challenging from the reaction selectivity point of view. In contrast to oxidative and redox-neutral dimerization, the reductive coupling can be tail-to-tail, head-to-tail, and head-to-head to yield 2,3-, 1,3-, and 1,4-dibranched-1,3-butadienes, respectively (Scheme 1). These reactions are mediated by transition metal complexes; tail-to-tail products have been achieved in the presence of palladium(0) species,^5^ while reactions of α,β-disubstituted titanacyclopentadiene compounds with protic reagents have provided head-to-tail couplings,^6^ and some zirconium,^7^ ruthenium,^8^ cobalt,^9^ and copper^10^ derivatives and palladium based MOFs^11^ have proven useful for realizing head-to-head assembly.

**Scheme 1:**
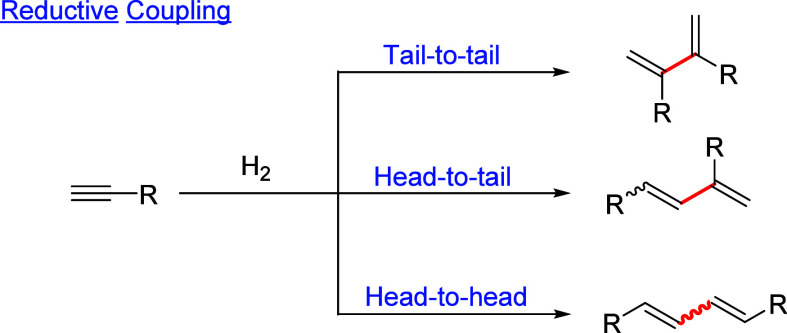
Reductive Dimerization of Terminal Alkynes

Terminal alkynes are also of great relevance in the synthesis of organometallic compounds.^12^ Their organic reactions developed in the coordination sphere of the transition metal complexes generate different classes of ligands, which stabilize a variety of organometallic derivatives. Such species are in many cases catalytic models or even real intermediates of metal-promoted organic synthesis reactions involving alkynes.^13^ Among the most promising organometallic precursors are the polyhydrides L_n_MH_*x*_ (*x* ≥ 3). This is a family of transition metal complexes with a very versatile chemistry,^14^ which gives them special relevance in catalysis^15^ and as starting compounds in developing challenging procedures to prepare organometallic derivatives of interest in materials science.^16^ Several reasons explain this versatility; MH_*x*_ units can contain three different types of ligands, which have diverse chemical properties: Kubas-type dihydrogen, elongated dihydrogen, and hydride.^14b,17^ Furthermore, these ligands may be in equilibrium in solution or undergo interconversion during a sequence of reactions.^14b,17,18^ This diversity results in polyhydride complexes that carry out homolytic and heterolytic σ-bond activation reactions. In fact, complexes with classical basic hydrides promote heterolytic cleavages; those that are saturated first release H_2_ through Kubas-type dihydrogen ligands to produce unsaturated species, which are then involved in homolytic cleavage.^14b^ Reactions of polyhydride complexes with terminal alkynes are a proof of the chemical versatility of those metal derivatives. Some unsaturated polyhydride compounds activate the C­(sp)–H bond of alkynes to give rise to alkynyl derivatives, which are able to act as efficient catalysts in the redox-neutral dimerization of such substrates.^19^ In other cases, alkynes generate monodentate C-donor ligands, which bind to the metal through a single,^20^ double^21^ or triple bond,^22^ depending on the alkyne substituent, the nature of the metal center, the number of hydrogen atoms in the MH_*x*_ unit, and the type of the other coligands. Some of the ligands generated in this way are the key point to achieve exciting aromatic metallacycles.^23^ There are also polyhydride complexes that reduce the C–C triple bond of alkynes.^24^

The OsH_6_(P^i^Pr_3_)_2_ complex is a prominent member of the polyhydride family,^25^ which is proving to be a cornerstone in the development of osmium organometallics due to its impressive stoichiometric and catalytic reactivity. Although it is a saturated species, at temperatures above 50 °C, it releases H_2_ to yield the unsaturated tetrahydride OsH_4_(P^i^Pr_3_)_2_, which has been trapped with a variety of Lewis bases.^26^ Through this tetrahydride, the hexahydride activates a wide range of σ-bonds,^27^ promotes metathesis between E–C­(sp^n^) and H–C­(sp^3^) bonds (E = Si, Ge; n = 2, 3),^28^ allows the development of alternative procedures for the preparation of osmium­(II) and osmium­(IV) phosphorescent emitters,^16,29^ and catalyzes a variety of classical organic transformations.^15^ Because complex OsH_6_(P^i^Pr_3_)_2_ generates a weakly reducing atmosphere, we were interested in exploring its reactivity toward phenylacetylene-type alkynes, in search of information on the capacity of osmium-polyhydride derivatives to carry out the reductive dimerization of terminal alkynes and to obtain evidence on the elementary steps that could comprise the process.

This paper presents the identification and characterization of reaction products between this hexahydride osmium complex and alkynes, under different experimental conditions, and rationalizes their formation by DFT calculations.

## Results and Discussion

### Reductive Head-to-Head Coupling of Phenylacetylenes

Tetrahydride OsH_4_(P^i^Pr_3_)_2_ (**A**) promotes the head-to-head coupling of phenylacetylenes and the monoreduction of the resulting organic entity, while the ensuing 1,4-dibranched-1,3-butadienes stabilize the generated metal-dihydride fragment (Scheme 2). Thus, treatment of solutions of its hexahydride synthon OsH_6_(P^i^Pr_3_)_2_ (**1**), in toluene, with 3.0 equiv of phenylacetylene, 4-(trifluoromethyl)­phenylacetylene and 4-(dimethylamino)­phenylacetylene, at 50 °C, for 14 h produces complexes OsH_2_{η^4^-[C_4_H_4_R_2_]}­(P^i^Pr_3_)_2_ (R = C_6_H_5_(**2**), C_6_H_4_–CF_3_(**3**), C_6_H_4_–NMe_2_(**4**)). Compounds **2** and **3** were isolated as white solids in high yield (80–85%). In contrast, complex **4** was obtained as a brown solid in a moderate yield of about 40%, due to a significant decomposition during the reaction, manifested by the additional formation of the saturated tetrahydride OsH_4_(P^i^Pr_3_)_3_ (**5**).

**Scheme 2:**
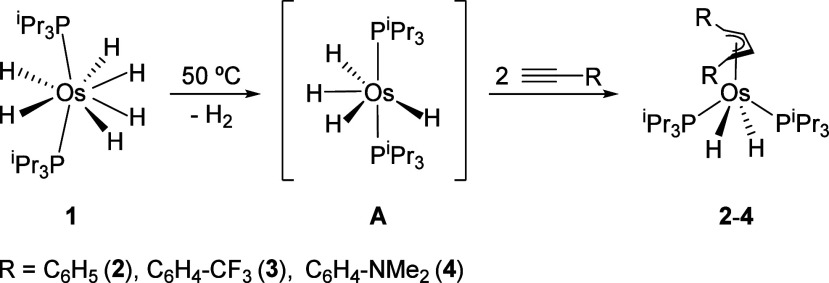
Formation of Complexes **2**−**4**

Complex **2** was characterized by X-ray diffraction analysis. Figure 1 shows a view of the molecular structure, which demonstrates the head-to-head reductive dimerization of the alkyne. The generated organic entity can be viewed as a planar^30^ 1,4-dibranched-butenediyl ligand, with three essentially similar C–C distances, which lie in the range of 1.441(2)−1.428(2) Å (1.436–1.428 Å in the DFT-optimized structure (B3LYP-D3//SDD-(f)/6-31-G**)) and are consistent with a bond order between the atoms of approximately 1.6. In this way, the distribution of donor atoms around the metal center can be rationalized as a four-legged piano stool, with the butenediyl on the seat and the hydride and phosphine ligands occupying the legs, alternately, forming P(1)–Os−P(2) and H(01)–Os−H(02) angles of 121.26(1)° and 115.5(10)°, respectively (123.6° and 115.4° in the DFT-optimized structure).This ligand arrangement is typical of half-sandwich derivatives of osmium­(IV), including the [OsH_2_(η^5^-C_5_R_5_)­(PR_3_)_2_]^+^ cations.^31^ The butenediyl coordination highlights the steric hindrance exerted by the phenyl substituents on the OsH_2_(P^i^Pr_3_)_2_ fragment. Thus, the terminal atoms C(1) and C(4) are slightly further from the metal center than C(2) and C(3); the Os–C(1) and Os–C(4) distances (2.265(1) and 2.314(1) Å; 2.314 and 2.371 Å in the DFT-optimized structure) are between 0.08 and 0.13 Å longer than the Os–C(2) and Os–C(3) lengths (2.180(1) and 2.186(1) Å; 2.207 and 2.213 Å in the DFT-optimized structure).

**Figure 1:**
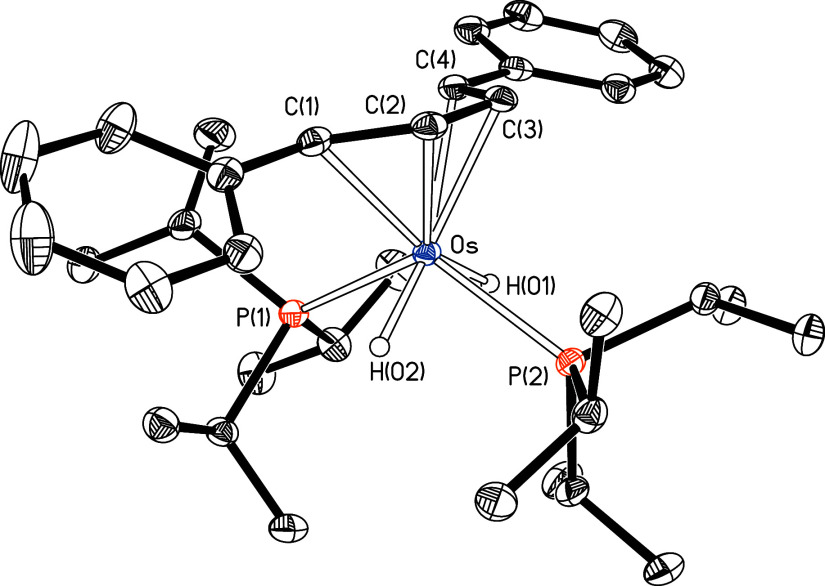
Molecular structure of **2** with ellipsoids at 50% probability level. Hydrogen atoms have been omitted for clarity, except hydride ligands. Selected bond distances (Å) and angles (deg): C(1)–C(2) = 1.441(2), C(2)–C(3) = 1.428(2), C(3)–C(4) = 1.432(2), Os–C(1) = 2.265(1), Os–C(2) = 2.180(1), Os–C(3) = 2.186(1), Os–C(4) = 2.314(1); P(1)–Os–P(2) = 121.26(1) and H(01)–Os–H(02) = 115.5(10).

The NMR characteristics of complexes **2**−**4**, in benzene-*d*_6_, at room temperature are consistent with the structure in Figure 1. In the ^1^H NMR spectra, the hydride ligands give rise to a doublet of doublets at approximately −14.1 ppm, with two different H–P coupling constants in the range 30–37 Hz, supporting the presence of two inequivalent phosphines *cisoid*-arranged to the hydrides. In the downfield region, the C_4_H_4_−butenediyl entity generates two signals, one near 5 ppm and the other between 1.6 and 1.2 ppm, which correlate with resonances at approximately 68 and 51 ppm in the ^13^C­{^1^H} NMR spectra. The inequivalent phosphines cause a second-order AB spin system in the ^31^P­{^1^H} NMR spectra, around 26 ppm, defined by *J*_AB_ in the range 82–91 Hz and Δν between 311 and 335 Hz.

To understand the head-to-head reductive dimerization process, we performed DFT calculations (SMD-(toluene)-B3LYP-D3//SDD-(f)/6-31-G**) using phenylacetylene as a model alkyne. The free energy changes (Δ*G*) were calculated at 298.15 K and 1 atm. Figure 2 shows the calculated energy profile, while Scheme 3 contextualizes the intermediates by sequencing the elementary reactions. The Cartesian coordinates of the optimized intermediates and transition states can be found in the xyz supplementary file.

**Figure 2:**
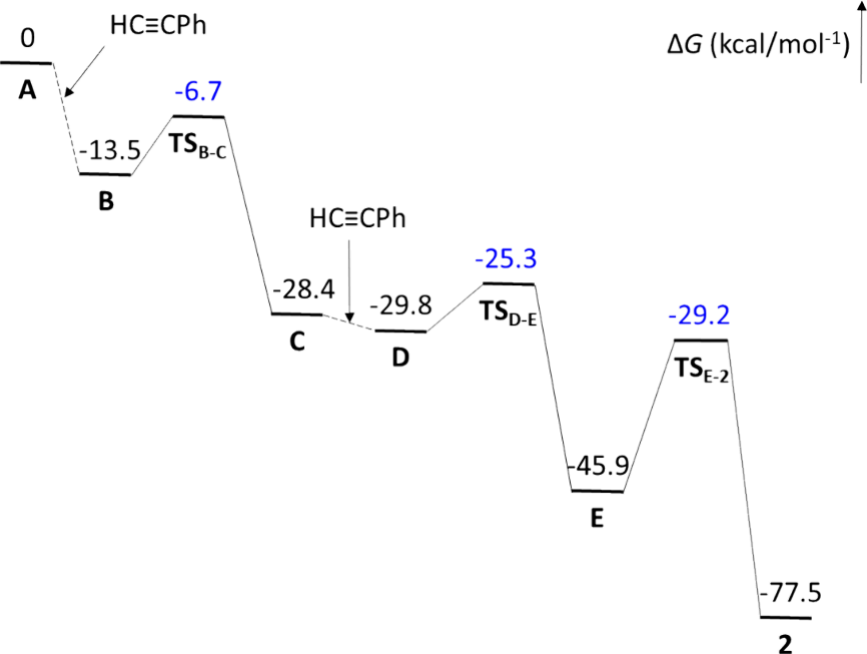
Energy profile (Δ*G*, in kcal mol^–1^) for the formation of **2**.

**Scheme 3:**
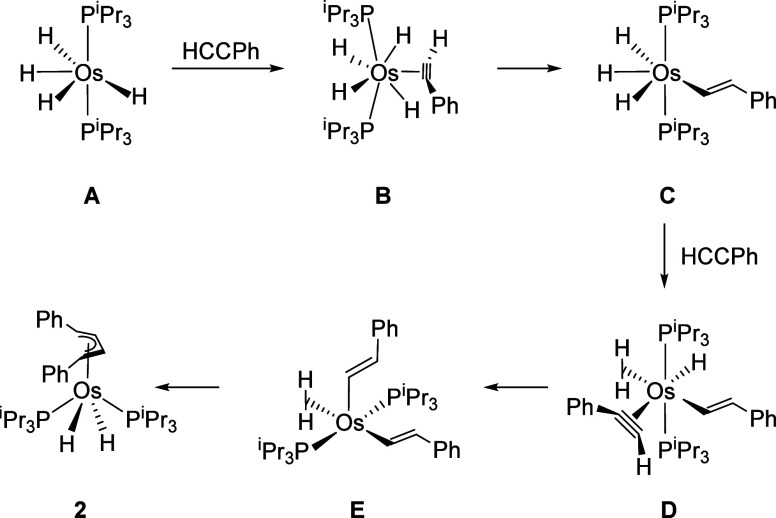
Theoretical Intermediate Steps for the Mechanism of the Formation of **2**

The formation of butenediyl ligand involves: the insertion of an alkyne molecule into an Os–H bond, the insertion of a second alkyne molecule into another Os–H bond, and the coupling of the resulting alkenyl groups (Scheme 3).

The first insertion takes place through the π-alkyne intermediate **B**, resulting from the coordination of the C–C triple bond of phenylacetylene, perpendicular to the P–Os–P direction of tetrahydride **A**. The coordination seems to be very easy (Figure S1), it has a very low activation energy of 1.4 kcal mol^–1^ and stabilizes the tetrahydride at 13.5 kcal mol^–1^. It occurs in three steps: coordination of the C­(sp)-H bond of the alkyne parallel to the P–Os–P direction of **A**, sliding of the metal center to the C–C triple bond, and 90° rotation of the coordinated bond. Intermediate **B** exhibits a dodecahedral structure defined by two intersecting orthogonal trapezoidal planes (P,H,H,P and C,C,H,H), with the substituted atom of the triple bond being the closest to the intersection. Approaching one of the hydride ligands in the P,H,H,P plane to the latter generates the typical four-center transition state, leading to intermediate **C**. The migration requires a low activation energy of 6.8 kcal mol^–1^ and produces an additional stabilization of 14.9 kcal mol^–1^.

Intermediate **C** arranges its ligand donor atoms in a trigonal antiprism around the metal center, similar to that of tetrahydride **A**, with the alkenyl group in the position of one of the hydrides. As in **A**, the unsaturated metal center of **C** coordinates the C–C triple bond of a new alkyne molecule, also perpendicular to the P–Os–P direction, to give **D**, which is 1.4 kcal mol^–1^ more stable than **C**. The structure of **D** resembles that of **B**, with the alkenyl group occupying the position of the hydride, neighboring to the unsubstituted atom of the triple bond, and two hydrides in the P,H,H,P plane forming a Kubas-type dihydrogen ligand (d_H–H_ = 0.916 Å). As a consequence of this arrangement, the C–C triple bond can in principle undergo two different insertions, resulting from the migration of the alkenyl group to the unsubstituted atom, to give a butadienyl derivative ^**1**^ **D** (Figure S2), and the migration of one of the hydrogen atoms from the dihydrogen ligand to the substituted atom, to produce the bis­(alkenyl)-compound **E**. Although ^**1**^ **D** is 4.4 kcal mol^–1^ more stable than **E**, the first insertion should also overcome an activation energy 17.4 kcal mol^–1^ higher than that of the second one. Therefore, the lower barrier for the migration of the hydrogen atom from the dihydrogen ligand to the substituted atom of the triple bond makes this route the preferred one. The reason could be associated with the lower steric requirement of the dihydrogen ligand compared to the alkenyl group and the lower directionality of the s orbital concerning the sp^2^-hybrid. Hydrogen migration overcomes a barrier of 4.5 kcal mol^–1^ to give intermediate **E**, which is 16.1 kcal mol^–1^ more stable than **D**. The process of formation of **E** can be understood as a concerted double migration of hydrogen, which occurs through a five-center transition state **TS**_**D‑E**_ resulting from the approach of the hydride to one of the hydrogen atoms of the dihydrogen and the approach of the other hydrogen atom of the dihydrogen to the substituted carbon atom of the C–C triple bond.

Intermediate **E** is a five-coordinate unsaturated bis­(alkenyl)-osmium­(II)-(Kubas-type dihydrogen) species (d_H–H_ = 0.878 Å), where the ligand donor atoms around the metal center define a square pyramid with one alkenyl group at the base and the other at the apex. In this context, it is worth remembering the marked tendency of this class of compounds to generate C–C bonds by axial-apical coupling.^32^ Consequently, the alkenyl groups couple to give the experimental complex **2**, through a three-center transition state, which is located 16.7 kcal mol^–1^ above **E**. The coupling is accompanied by the breaking of the H–H bond of the dihydrogen ligand, which causes the oxidation of the metal center. The overall reaction starting from hexahydride **1** is an exergonic process by 68.6 kcal mol^–1^; 77.5 kcal mol^–1^ with respect to **A**.

### Head-to-Head Reductive Coupling of Phenylacetylenes with Dehydrogenations of the Metal and Alkylphosphine

The reactions summarized in Scheme 2 are very sensitive to temperature and the **1**:alkyne molar ratio. An increase in the amount of the tetrahydride complex **5** is observed, along with darkening of the solution and a decrease in the yield of **2**−**4** formation, with increasing reaction temperatures, for **1**:alkyne molar ratios greater than 1:3. A gradual increase in the amount of alkyne added to the reaction produces a gradual reduction in the degree of decomposition and formation of **5**, but a new class of organometallic species arises. These complexes were characterized as Os­[η^4^-(C_4_H_4_R_2_)]­{η^2^-*C,C*;κ^1^-*P*-[(CH_2_=CMe)­P^i^Pr_2_]}­(P^i^Pr_3_) (R = C_6_H_5_(**6**), C_6_H_4_–CF_3_(**7**), C_6_H_4_–NMe_2_(**8**)). Their amounts depend on the phenyl group substituents, increasing in the sequence Me_2_N < H < CF_3_. At 125 °C, using 5 equiv of alkyne, the respective butenediyl derivatives **2**−**4** and **6**−**8** are the only organometallic compounds detected in each reaction mixture, in the relative amounts given in Scheme 4.^33^ The formation of **6**−**8** reveals that the head-to-head reductive coupling of the alkyne can be accompanied by two dehydrogenation reactions, without undergoing any alteration; one at the metal center and the other involving an isopropyl substituent of a phosphine.^34^ The hydrogen acceptor for the dehydrogenation processes is the alkyne itself. In agreement with this, the respective styrenes were the main organic products of the reactions.

**Scheme 4:**
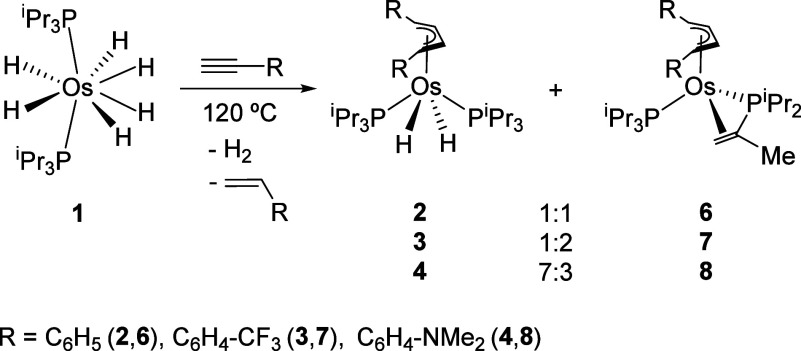
Formation of **2**−**4** and **6−8** Derivatives

The strongest evidence for the existence of **6**−**8** comes from the X-ray diffraction analysis structure of **7**.^35^ The structure has two chemically equivalent but crystallographically independent molecules in the asymmetric unit; Figure 3 shows one of them. The coordination around the metal center is as expected for a *d* ^*6*^ ion and can be rationalized as a distorted octahedron. The butenediyl ligand occupies all three sites on one face, showing similar features to those observed in **2**; the C–C distances are in the range 1.453(6)−1.414(6) Å and the terminal atoms C(1) and C(4) are also slightly farther from the metal center than the central atoms C(2) and C(3) (2.293(4)−2.250(4) and 2.291(4)−2.255(4) Å versus 2.153(4)−2.140(4) and 2.158(4)−2.140(4) Å). The midpoint (M) of the C(19)–C(20) coordinated double bond (1.435(5) and 1.436(6) Å) of the isopropenyl substituent, generated at one of the phosphines, and the phosphorus atoms are located in the sites of the opposite face, which suffers a strong distortion due to the ring restriction imposed by the bidentate phosphine (P(1)–Os(1)−-M = 57.6(1)° and 57.4(1)°)).

**Figure 3:**
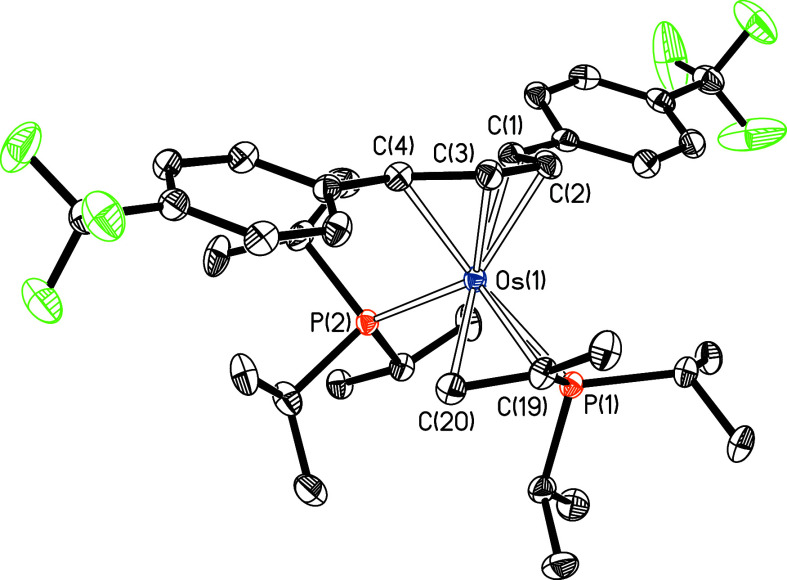
Molecular structure of **7** with ellipsoids at 50% probability level. Hydrogen atoms have been omitted for clarity. Selected bond distances (Å) and angles (deg): C(1)–C(2) = 1.444(5), 1.453(6), C(2)–C(3) = 1.425(6), 1.414(6), C(3)–C(4) = 1.433(5), 1.442(6), C(19)–C(20) = 1.435(5), 1.436(6), Os–C(1) = 2.293(4), 2.291(4), Os–C(2) = 2.153(4), 2.158(4), Os–C(3) = 2.140(4), 2.140(4), Os–C(4) = 2.250(4), 2.255(4); P(1)–Os–P(2) = 99.91(3), 100.64(3), and P(2)–Os–M = 57.6(1), 57.4(1).

The NMR spectra of **6**−**8**, in benzene-*d*_6_, at room temperature are consistent with the structure in Figure 3. In agreement with the asymmetry of these molecules, the ^13^C­{^1^H} NMR spectra show four resonances between 72 and 45 ppm due to the inequivalent carbon atoms of the butenediyl skeleton, in addition to the signals corresponding to the coordinated atoms of the isopropenyl group around 23 (PC) and 18 (CH_2_) ppm. The ^31^P­{^1^H} spectra contain two doublets at approximately 18 (P^i^Pr_3_) and −55 (P^i^Pr_2_[C­(CH_3_)=CH_2_]) ppm with a P–P coupling constant of around 25 Hz.

One might in principle think that complexes **6**−**8** are generated by hydrogen transfer from **2**−**4** to molecules of the respective alkynes, present in excess in the reactions. However, solutions of **2**−**4**, in toluene, at 120 °C remain unchanged after stirring in the presence of 5.0 equiv of the alkyne, for 24 h. Other hydrogen acceptors such as cyclohexene or tetramethylethylene also do not promote any of the dehydrogenations. This reveals that the formations of **2**−**4** and **6**–**8** are not sequential but competitive processes. In other words, the head-to-head reductive coupling of the alkynes and the dehydrogenations are independent processes, although the participation of some styryl intermediate common to all of them cannot be ruled out.

### Reactions with Hydrogen

Both families of complexes **2**−**4** and **6**−**8** react with molecular hydrogen to regenerate hexahydride **1**, liberating the corresponding 1,4-diaryl-butane hydrogenated products. The difference in the hydrogenation rates of related members of each family does not appear to be significant. In agreement with this, we observed that a mixture of **3** and **7** generated in toluene-*d*_*8*_ evolved to **1** under 1 atm of H_2_, after 20 h, at 90 °C, maintaining a similar molar ratio between the complexes in the mixture until the end (Figure S3). Because the difference in stoichiometry between **2**−**4** and **6**−**8** is two molecules of H_2_, this suggests that the hydrogenation of **2**−**4** is independent of that of **6**−**8**. That also supports the idea that complexes **2**−**4** are not intermediate species in the hydrogenation of **6**−**8**. Thus, the independent formation reactions of **2**−**4** and **6**−**8** and the independent hydrogenation reactions of **2**−**4** and **6**−**8** constitute two metal-promoted stoichiometric organic synthesis cycles for the head-to-head reductive dimerization of phenylacetylenes to 1,4-diarylbutanes (Scheme 5). The one-pot procedure for preparing these compounds usually involves lithium-promoted reductive dimerization of styrenes.^36^

**Scheme 5:**
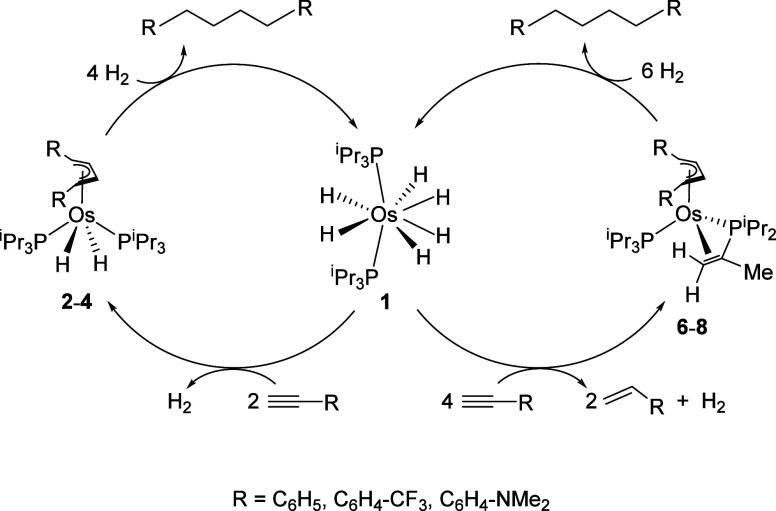
Stoichiometric Cycles for the Reductive Head-to-Head Coupling of Phenylacetylenes Promoted by **1**, through Complexes **2**−**4** and **6**−**8**

To ascertain the catalytic utility of these cycles, we stirred solutions of phenylacetylene and 4-(trifluoromethyl)­phenylacetylene, in toluene, with amounts of hexahydride complex **1** in the range 2–10 mol %, between 50 and 90 °C, for 24 h, under 1 atm of H_2_, and in the presence of dioxane as standard. Under these conditions, complex **1** mainly promotes the complete hydrogenation of the C–C triple bond of alkynes to produce ethylbenzenes. However, variable amounts of 1,4-diaryl-butanes were also observed, depending on the alkyne:osmium molar ratio used and the temperature. Maximum amounts of alkyne of approximately 20% were transformed into the respective 1,4-diaryl-butanes using 5 mol % of **1** and 90 °C. Therefore, although hexahydride **1** is primarily an active catalyst for the reduction of the C–C triple bond of phenylacetylenes under a hydrogen atmosphere, these results show that it can also promote the head-to-head reductive dimerization of this type of alkynes to give 1,4-diaryl-butanes.

## Concluding Remarks

This study has revealed that the hexahydride complex OsH_6_(P^i^Pr_3_)_2_ promotes the head-to-head reductive coupling of phenylacetylenes to provide the 1,4-dibranched-butenediyl derivatives OsH_2_[η^4^-(C_4_H_4_R_2_)]­(P^i^Pr_3_)_2_, via the tetrahydride specie OsH_4_(P^i^Pr_3_)_2_. The results of the DFT calculations indicate that the process involves the sequential insertions of two alkyne molecules into Os–H bonds of the tetrahydride intermediate to generate a five-coordinate unsaturated bis­(alkenyl)-osmium­(II)-(Kubas-type dihydrogen), which evolves by alkenyl coupling and H–H scission of the dihydrogen ligand. Under a hydrogen atmosphere, complexes OsH_2_[η^4^-(C_4_H_4_R_2_)]­(P^i^Pr_3_)_2_ regenerate the hexahydride OsH_6_(P^i^Pr_3_)_2_, releasing the respective 1,4-diaryl-butanes. Consistently, the preparation of these organic molecules can be achieved with a yield of approximately 20%, by dimerization of phenylacetylenes, in the presence of 5 mol % of the hexahydride, under a hydrogen atmosphere.

Further work is needed to achieve a satisfactory level of development, but in light of these results, it is clear that polyhydride complexes are promising promoters of reductive couplings of terminal alkynes, with potential utility in organometallic and organic synthesis.

## Experimental Section

### General Information

All reactions were performed with rigorous exclusion of moisture and air, using an argon/vacuum manifold and standard Schlenk-tube or glovebox techniques. Complex **1** was prepared according to the published methods.^25^ Alkynes were purchased from commercial sources and distilled prior to use. Instrumental methods, X-ray, theoretical calculations details, and NMR spectra (Figures S1–S33) are given in the Supporting Information. Coupling constants *J* and Δυ are given in hertz.

### Preparation of OsH_2_{η^4^-[C_4_H_4_(C_6_H_5_)_2_]}­(P^i^Pr_3_)_2_ (2)

Phenylacetylene (160 μL, 0.87 mmol) was added to a solution of **1** (150 mg, 0.29 mmol), in toluene (10 mL). Then, the mixture was heated at 50 °C, for 14 h. After that, the solution was evaporated to dryness to give a brown oil, which was treated four times with 4 mL of pentane. The resulting solutions were collected together and concentrated to dryness. The residue was stirred in 4 mL of methanol at −78 °C until a pale beige solid was formed. Yield: 170 mg (80%). White single crystals suitable for X-ray diffraction analysis were obtained from a saturated solution of **2** in pentane. Anal. Calcd for C_34_H_58_OsP_2_: C, 56.80; H, 8.13. Found; C, 56.78; H, 8.11. HRMS (electrospray, *m*/*z*) calcd. for C_34_H_57_OsP_2_ [M-H]^+^: 719.3547; found: 719.3577. IR (cm^–1^): ν­(Os–H) 2076. ^1^H NMR (300.13 MHz, C_6_D_6_, 298 K): δ 7.43–7.01 (10H, C_6_H_5_), 5.14 (br, 2H, C_4_H_4_), 2.31–2.21 (m, 3H, PC*H*(CH_3_)_2_), 2.17–2.07 (m, 3H, PC*H*(CH_3_)_2_), 1.46 (br, 2H, C_4_H_4_), 1.06 (dd, ^3^ *J*_H–P_ = 12.7, ^3^ *J*_H–H_ = 7.1, 18H, PCH­(C*H*_3_)_2_), 0.98 (dd, ^3^ *J*_H–P_ = 12.7, ^3^ *J*_H–H_ = 7.1, 18H, PCH­(C*H*_3_)_2_), −14.12 (dd, ^2^ *J*_H–P_ = 35.9, 30.7, 2H, OsH_2_). ^31^P­{^1^H} NMR (121.50 MHz, C_6_D_6_, 298 K): δ 26.5 (AB system, *J*_AB_ = 85.0 Hz; Δυ = 318 Hz). ^13^C­{^1^H}-APT NMR (75.48 MHz, C_6_D_6_, 298 K): δ 147.7 (s, C_arom_), 128.0, 127.3, 123.7 (s, CH_arom_), 68.8 (br, C_4_H_4_), 51.6 (dd, ^2^ *J*_C–P_ = 7.6, 5.6, C_4_H_4_), 31.9 (d, ^1^ *J*_C–P_ = 26.7, P*C*H­(CH_3_)_2_), 30.2 (d, ^1^ *J*_C–P_ = 23.8, P*C*H­(CH_3_)_2_), 20.4, 19.8 (s, PCH­(*C*H_3_)_2_).

### Preparation of OsH_2_{η^4^-[C_4_H_4_(C_6_H_4_−CF_3_)_2_]}­(P^i^Pr_3_)_2_ (3)

Complex **1** (150 mg, 0.29 mmol) was dissolved in toluene (10 mL), and then 4-(trifluoromethyl)­phenylacetylene (140 μL, 0.87 mmol) was added to the solution. The resulting mixture was heated at 50 °C for 14 h. After that time, the solution was evaporated to dryness to give a dark brown oil. Adding MeOH (3 mL) at −78 °C, a white solid appeared, which was washed with cold MeOH (4 × 3 mL). Yield: 210 mg (85%). Anal. Calcd for C_36_H_56_F_6_OsP_2_: C, 50.57; H, 6.60. Found; C, 50.31; H, 6.28. HRMS (electrospray, *m*/*z*) calc. for C_36_H_55_F_6_OsP_2_ [M-H]^+^: 855.3297; found: 855.3280. IR (cm^–1^): ν­(Os–H) 2158. ^1^H NMR (300.13 MHz, C_6_D_6_, 298 K): δ 7.43 (d, ^3^ *J*_H–H_ = 8.1, 4H, C_6_H_4_–CF_3_), 7.22 (d, ^3^ *J*_H–H_ = 8.1, 4H, C_6_H_4_–CF_3_), 5.03 (br, 2H, C_4_H_4_), 2.13–1.97 (m, 6H, PC*H*(CH_3_)_2_), 1.25 (br, 2H, C_4_H_4_), 0.97 (dd, ^3^ *J*_H–P_ = 12.9, ^3^ *J*_H–H_ = 7.1, 18H, PCH­(C*H*_3_)_2_), 0.84 (dd, ^3^ *J*_H–P_ = 12.9, ^3^ *J*_H–H_ = 7.1, 18H, PCH­(C*H*_3_)_2_), −14.17 (dd, ^2^ *J*_H–P_ = 35.6, 30.9, 2H, OsH_2_). ^31^P­{^1^H} NMR (121.50 MHz, C_6_D_6_, 298 K): δ 26.4 (AB system, *J*_AB_ = 82.0 Hz; Δυ = 311 Hz). ^13^C­{^1^H}-APT NMR (75.48 MHz, C_6_D_6_, 298 K): δ 152.1 (s, C_arom_), 130.9 (inferred from HMBC spectrum, C_arom_), 127 (s, CH_arom_), 124.9 (br, CH_arom_), 120.1 (inferred from HMBC spectrum, *C*F_3_), 69.3 (dd, ^2^ *J*_C–P_ = 3.1, 3.1, C_4_H_4_), 50.3 (br, C_4_H_4_), 31.8 (d, ^1^ *J*_C–P_ = 27, P*C*H­(CH_3_)_2_), 30.2 (d, ^1^ *J*_C–P_ = 24.2, P*C*H­(CH_3_)_2_), 20.3, 19.5 (s, PCH­(*C*H_3_)_2_). ^19^F­{^1^H} NMR (282.38 MHz, C_6_D_6_, 298 K): δ −61.43.

### Preparation of OsH_2_{η^4^-[C_4_H_4_(C_6_H_4_−NMe_2_)_2_]­(P^i^Pr_3_)_2_ (4)

A Schlenk tube was charged with **1** (150 mg, 0.29 mmol), toluene (10 mL), and 4-(dimethylamine)­phenylacetylene (125 mg, 0.87 mmol) and heated at 50 °C for 14 h. After that time, the solution was concentrated to the formation of a dark brown oil, which was treated four times with 4 mL of pentane. The resulting solutions were collected together and concentrated to dryness. The residue was stirred in methanol (3 mL), at −78 °C, to afford a pale yellow solid, which was washed with cold methanol (4 × 3 mL) and vacuum drying. Yield: 96 mg (41%). Anal. Calcd for C_38_H_68_N_2_OsP_2_: C, 56.69; H, 8.51; N, 3.48. Found; C, 56.75; H, 8.62; N, 3.56. HRMS (electrospray, *m*/*z*) calc. for C_38_H_68_N_2_OsP_2_Na [M + Na]^+^: 829.4370; found: 829.4398. IR (cm^–1^): ν­(Os–H) 2162. ^1^H NMR (300.13 MHz, C_6_D_6_, 298 K): δ 7.45 (d, ^3^ *J*_H–H_ = 8.6, 4H, C_6_ *H*_4_−NMe_2_), 6.73 (d, ^3^ *J*_H–H_ = 8.8, 4H, C_6_ *H*_4_−NMe_2_), 5.12 (br, 2H, C_4_H_4_), 2.59 (s, 12H, N*Me*_2_), 2.27–2.13 (m, 6H, PC*H*(CH_3_)_2_), 1.61 (br, 2H, C_4_H_4_), 1.16 (dd, ^3^ *J*_H–P_ = 12.5, ^3^ *J*_H–H_ = 7.1, 18H, PCH­(C*H*_3_)_2_), 1.09 (dd, ^3^ *J*_H–H_ = 12.5, ^3^ *J*_H–H_ = 7.1, 18H, PCH­(C*H*_3_)_2_), −14.15 (dd, ^2^ *J*_H–P_ = 36.1, 30.5, 2H, OsH_2_). ^31^P­{^1^H} NMR (121.50 MHz, C_6_D_6_, 298 K): δ 26.9 (AB system, *J*_AB_ = 91.2 Hz; Δυ = 335 Hz). ^13^C­{^1^H}-APT NMR (75.48 MHz, C_6_D_6_, 298 K): δ 148.0, 136.4 (s, C_arom_), 131.6, 130.9, 130.4, 126.8, 113.2 (s, CH_arom_), 67.7 (br, C_4_H_4_), 52.0 (br, C_4_H_4_), 40.9 (s, NMe_2_), 31.9 (d, ^1^ *J*_C–P_ = 25.6, P*C*H­(CH_3_)_2_), 30.3 (d, ^1^ *J*_C–P_ = 23.7, P*C*H­(CH_3_)_2_), 20.6, 20.1 (s, PCH­(*C*H_3_)_2_).

### Characterization of OsH_4_(P^i^Pr_3_)_3_ (5)

NMR spectra of the residue obtained upon evaporation of discarded methanol solution from complex **4** workup showed the presence of complexes **4** and **5** in a ratio 1:4. HRMS (electrospray, *m*/*z*) calcd. for C_27_H_65_OsP_3_ [M-2H]^+^: 674.3908; found: 674.3899. NMR Features for **5**: ^1^H NMR (300.13 MHz, C_6_D_6_, 298 K): δ 2.04–1.92 (9H, PC*H*(CH_3_)_2_), 1.28–1.22 (54H, PCH­(C*H*_3_)_2_), −11.65 (q, ^2^ *J*_H–P_ = 9.6, OsH_4_). ^31^P­{^1^H} NMR (121.50 MHz, C_6_D_6_, 298 K): δ 32.1 (s). ^13^C­{^1^H}-APT NMR (75.48 MHz, C_6_D_6_, 298 K): δ 31.2–30.9 (P*C*H­(CH_3_)_2_), 20.7 (s, PCH­(*C*H_3_)_2_). Attempts to stop the fluxionality of the complex were unsuccessful even upon cooling the sample up to 183 K. At this temperature, only a slight broadening of the resonances was observed.

### Formation and Characterization of Os­{η^4^-[C_4_H_4_(C_6_H_5_)_2_]}­{η^2^-*C,C*;κ^1^-*P*-[(CH_2_=CMe)­P^i^Pr_2_]}­(P^i^Pr_3_) (6)

Phenylacetylene (105 μL, 0.95 mmol) was added to a solution of **1** (100 mg, 0.19 mmol), in toluene (15 mL). The mixture was heated at 125 °C, for 6 h. After that time, the solution was evaporated to dryness to give a dark orange oil. Its NMR spectra in benzene-*d*_6_ revealed the presence of complexes **2** and **6** in a 1:1 molar ratio. Yellow single crystals of **6** were obtained from a saturated cold solution of a mixture of **2** and **6** in pentane. HRMS (electrospray, *m*/*z*) calcd. for C_34_H_54_OsP_2_Na [M + Na]^+^: 739.3213; found: 739.3228. ^1^H NMR (300.13 MHz, C_6_D_6_, 298 K): δ 7.40–7.03 (10H, C_6_H_5_), 5.07 (br, 1H, C_4_H_4_), 4.26 (br, 1H, C_4_H_4_), 2.39–2.27 (m, 3H, PC*H*(CH_3_)_2_), 1.98–1.86 (m, 1H, PC*H*(CH_3_)_2_), 1.83–1.79 (m, 1H, PC=CH_2_), 1.71 (dd, ^3^ *J*_H–P_ = 6.7, ^5^ *J*_H–P_ = 2.1, 3H, PCCH_3_), 1.53–1.48 (m, 1H, PC=CH_2_), 1.46 (br, 1H, C_4_H_4_), 1.29 (inferred from COSY spectrum, 1H, C_4_H_4_), 1.28 (dd, ^3^ *J*_H–P_ = 14.8, ^2^ *J*_H–H_ = 7.7, 3H, PCH­(C*H*_3_)_2_), 1.23 (dd, ^3^ *J*_H–P_ = 15.4, ^2^ *J*_H–H_ = 7.9, 3H, PCH­(C*H*_3_)_2_), 1.18 (m, 3H, PCH­(C*H*_3_)_2_), 1.15 (inferred from COSY spectrum, 1H, PC*H*(CH_3_)_2_), 1.12 (dd, ^3^ *J*_H–P_ = 11.5, ^3^ *J*_H–H_ = 7.3, 9H, PCH­(C*H*_3_)_2_), 1.01 (dd, ^3^ *J*_H–P_ = 13.2, ^3^ *J*_H–H_ = 7.3, 9H, PCH­(C*H*_3_)_2_), 0.85 (dd, ^3^ *J*_H–P_ = 15.2, ^2^ *J*_H–H_ = 7.4, 3H, PCH­(C*H*_3_)_2_).^31^P­{^1^H} NMR (121.50 MHz, C_6_D_6_, 298 K): δ 17.6 (d, ^2^ *J*_P–P_ = 25.5), −54.8 (d, ^2^ *J*_P–P_ = 25.5). ^13^C­{^1^H}-APT NMR (75.48 MHz, C_6_D_6_, 298 K): δ 150.3, 146.9 (s, C_arom_), 127.3, 126.9, 123.0 (s, CH_arom_), 71.8 (d, ^2^ *J*_C–P_ = 4.0, C_4_H_4_), 64.4 (d, ^2^ *J*_C–P_ = 4.2, C_4_H_4_), 47.5 (dd, ^2^ *J*_C–P_ = 24.4, ^2^ *J*_C–P_ = 4.6, C_4_H_4_), 46,7 (dd, ^2^ *J*_C–P_ = 6.4, ^2^ *J*_C–P_ = 2.5, C_4_H_4_), 31.1 (dd, ^1^ *J*_C–P_ = 13.7, ^3^ *J*_C–P_ = 1.9, P*C*H­(CH_3_)_2_), 30.6 (dd, ^1^ *J*_C–P_ = 20.9, ^3^ *J*_C–P_ = 3.2, P*C*H­(CH_3_)_2_), 27.8 (d, ^2^ *J*_C–P_ = 9.3, PCH­(*C*H_3_)_2_), 25.9 (d, ^2^ *J*_C–P_ = 4.6, P*C*H­(CH_3_)_2_), 23.1 (d, ^2^ *J*_C–P_ = 8.5, PCH­(*C*H_3_)_2_), 22.8 (d, ^1^ *J*_C–P_ = 8.0, P*C*=CH_2_), 21.8 (d, ^2^ *J*_C–P_ = 5.2, PCH­(*C*H_3_)_2_), 21.3 (br, PCH­(*C*H_3_)_2_), 20.3 (d, ^2^ *J*_C–P_ = 2.3, PCH­(*C*H_3_)_2_), 19.7 (d, ^2^ *J*_C–P_ = 3.0, PC­(*C*H_3_)), 18.8 (d, ^2^ *J*_C–P_ = 3.0, PCH­(*C*H_3_)_2_), 18.1 (br, PC=*C*H_2_).

### Formation and Characterization of Os­{η^4^-[C_4_H_4_(C_6_H_4_CF_3_)_2_]}­{η^2^-*C,C*;κ^1^-*P*-[(CH_2_=CMe)­P^i^Pr_2_]}­(P^i^Pr_3_) (7)

A Schlenk tube was changed with OsH_6_(P^i^Pr_3_)_2_ (100 mg, 0.19 mmol) and toluene (15 mL). 4-(trifluoromethyl)­phenylacetylene (155 μL, 0.95 mmol) was added to the solution and the mixture was heated at 125 °C for 6 h. After that time, it was evaporated to dryness to give a dark brown oil. Its NMR spectra in benzene-*d*_6_ revealed the presence of complexes **3** and **7** in a 1:2 molar ratio. Features for **7**: HRMS (electrospray, *m*/*z*) calcd. for C_36_H_52_F_6_OsP_2_ [M]^+^: 852.3063; found: 852.3036. ^1^H NMR (300.13 MHz, C_6_D_6_, 298 K): δ 7.52–7.34 (6H, C_6_H_4_–CF_3_), 6.91–6.85 (2H, C_6_H_4_–CF_3_) 4.50 (br, 1H, C_4_H_4_), 4.14 (br, 1H, C_4_H_4_), 2.22–2.09 (3H, PC*H*(CH_3_)_2_), 1.78–1.70 (1H PC*H*(CH_3_)_2_ + 1H PC=CH_2_), 1.60 (dd, ^3^ *J*_H–P_ = 6.8, ^5^ *J*_H–P_ = 1.8, 3H, PCCH_3_), 1.40–1.33 (1H C_4_H_4_ + 1H PC=CH_2_), 1.30–1.27 (m, 1H, PC*H*(CH_3_)_2_), 1.26–1.12 (9H, PCH­(C*H*_3_)_2_), 1.16 (inferred from COSY spectrum, 1H, C_4_H_4_), 1.01 (dd, ^3^ *J*_H–P_ = 11.9, ^2^ *J*_H–H_ = 7.2, 9H, PCH­(C*H*_3_)_2_), 0.82 (dd, ^3^ *J*_H–P_ = 13.5, ^2^ *J*_H–H_ = 7.3, 9H, PCH­(C*H*_3_)_2_), 0.74 (dd, ^3^ *J*_H–P_ = 15.6, ^2^ *J*_H–H_ = 7.5, 3H, PCH­(C*H*_3_)_2_). ^31^P­{^1^H} NMR (121.50 MHz, C_6_D_6_, 298 K): δ 17.2 (d, ^2^ *J*_P–P_ = 25.0), −55.1 (d, ^2^ *J*_P–P_ = 25.0). ^13^C­{^1^H}-APT NMR (75.48 MHz, C_6_D_6_, 298 K): δ 154.9, 151.2 (s, C_arom_), 126.6 (s, CH_arom_), 125.8 (br, CH_arom_), 71.9 (d, ^2^ *J*_C–P_ = 3.8, C_4_H_4_), 64.7 (d, ^2^ *J*_C–P_ = 3.9, C_4_H_4_), 46.6 (dd, ^2^ *J*_C–P_ = 24.4, ^2^ *J*_C–P_ = 4.4, C_4_H_4_), 45.8 (d, ^2^ *J*_C–P_ = 5.0, C_4_H_4_), 30.9 (d, ^1^ *J*_C–P_ = 15.8, P*C*H­(CH_3_)_2_), 30.4 (dd, ^1^ *J*_C–P_ = 20.9, P*C*H­(CH_3_)_2_), 27.6 (d, ^2^ *J*_C–P_ = 7.9, PCH­(*C*H_3_)_2_), 26.0 (d, ^2^ *J*_C–P_ = 6.0, P*C*H­(CH_3_)_2_), 23.9 (inferred from HMBC spectrum, P*C*=CH_2_), 22.9 (d, ^2^ *J*_C–P_ = 8.7, PCH­(*C*H_3_)_2_), 21.6 (d, ^2^ *J*_C–P_ = 4.9, PCH­(*C*H_3_)_2_), 20.9 (s, PCH­(*C*H_3_)_2_), 20.0 (s, PC­(*C*H_3_)), 18.6 (s, PCH­(*C*H_3_)_2_), 18.4 (inferred from HMBC spectrum, PC=*C*H_2_). ^19^F­{^1^H} NMR (282.38 MHz, C_6_D_6_, 298 K): δ −61.33.

### Formation and Characterization of Os­{η^4^-[C_4_H_4_(C_6_H_4_−NMe_2_)_2_]}­{η^2^-*C*,*C*;κ^1^-*P*-[(CH_2_=CMe)­P^i^Pr_2_]}­(P^i^Pr_3_) (8)

4-(dimethylamine)­phenylacetylene (140 mg, 0.95 mmol) was added to a solution of **1** (100 mg, 0.19 mmol) in toluene (15 mL). The mixture was heated at 125 °C, for 6 h. After that time, it was evaporated to dryness to give a dark brown oil. Its NMR spectra in benzene-*d*_6_ revealed the presence of complexes **4** and **8** in a 7:3 molar ratio. Features for **8**: HRMS (electrospray, *m*/*z*) calcd. for C_38_H_64_N_2_OsP_2_ [M]^+^: 802.4154; found: 802.4189. ^1^H NMR (300.13 MHz, C_6_D_6_, 298 K): δ 7.16 (d, ^3^ *J*_H–H_ = 8.6, 4H, C_6_ *H*_4_−NMe_2_), 6.59 (d, ^3^ *J*_H–H_ = 8.8, 4H, C_6_ *H*_4_−NMe_2_), 5.12 (br, 1H, C_4_H_4_), 4.26 (br, 1H, C_4_H_4_), 2.60 (s, 6H, N*Me*_2_), 2.52 (s, 6H, N*Me*_2_), 2.28–0.70 (21H P­[CH­(CH_3_)_2_]_3_ + 19H (CH_2_=C­(Me)­P­(CH­(CH_3_)_2_)_2_ + 2H C_4_H_4_). ^31^P­{^1^H} NMR (121.50 MHz, C_6_D_6_, 298 K): δ 18.1 (d, ^2^J_P–P_ = 25.2), −54.2 (d, ^2^J_P–P_ = 25.2).^13^C­{^1^H}-APT NMR (75.48 MHz, C_6_D_6_, 298 K): δ 150.1, 149.6, 132.7, 130.6 (s, C_arom_), 129.4, 128.7, 127.5, 126.2, 113.6, 113.5, 113.2, 113.1 (s, CH_arom_), 71.2 (d, ^2^J_C–P_ = 3.9, C_4_H_4_), 63.6 (d, ^2^ *J*_C–P_ = 3.8, C_4_H_4_), 48.1 (br, C_4_H_4_), 46,8 (d, ^2^ *J*_C–P_ = 3.8, C_4_H_4_), 40.7, 40.7, 40.3 (s, NMe_2_)), 31.1 (br, P*C*H­(CH_3_)_2_), 30.7 (d, ^1^ *J*_C–P_ = 17.3, P*C*H­(CH_3_)_2_), 27.9 (d, ^2^ *J*_C–P_ = 8.3, PCH­(*C*H_3_)_2_), 25.8 (br, P*C*H­(CH_3_)_2_), 23.1 (d, ^2^ *J*_C–P_ = 8.9, PCH­(*C*H_3_)_2_), 21.2 (br, PCH­(*C*H_3_)_2_), 21.7 (inferred from HMBC spectrum, P*C*=CH_2_), 21.0 (s, PCH­(*C*H_3_)_2_), 20.7 (br, PCH­(*C*H_3_)_2_),19.4 (br, PC­(*C*H_3_)), 17.3 (inferred from HMBC spectrum, PC=*C*H_2_).

### Reaction of the Mixture of Complexes 3 and 7 with H_2_

A pressure valve NMR tube was charged with a solution of **3** and **7** (3:2) in toluene (0.5 mL). The argon atmosphere was replaced by H_2_ (1 atm) and the mixture was heated at 90 °C for 20 h. After this time, the formation of **1** and 4-bis­(4-trifluoromethyl)-phenyl)­butane was observed on ^1^H and ^31^P­{^1^H} NMR spectroscopies.

### General Procedure for Reductive Coupling Catalytic Reactions

Inside an argon-filled glovebox, **1** (0.01 mmol) was dissolved in 0.5 mL of dried toluene-*d*_8_. Then, alkyne (0.4 mmol) and dioxane (0.01 mmol) were added to the solution. Outside the glovebox, the argon atmosphere was replaced by H_2_ (1 atm.). The reaction mixture was heated at 90 °C, for 24 h. NMR yields were calculated using ^1^H NMR experiments (d1 = 10 s). ^1^H NMR signals from the organic products obtained matched with the previously described in the bibliography.^37^

## Supplementary Material





## Notes

Experimental details, crystallographic data, theoretical studies, NMR and IR spectra (PDF)
Cartesian coordinates of the optimized structures (XYZ)
