# Supplementary material for: Reductive Head-to-Head Coupling of Phenylacetylenes in the Coordination Sphere of an Osmium-Polyhydride
Source: Organometallics. 2025 Jan 6;44(2):447–55. doi: 10.1021/acs.organomet.4c00480 (PMC12879066; doi:10.1021/acs.organomet.4c00480)
Supplement: Supplementary file 1 [file om4c00480_si_001.pdf]

Supporting Information

Reductive Head-to-Head Coupling of Phenylacetylenes in the Coordination Sphere of an Osmium-Polyhydride

Sheila G. Curto, Miguel A. Esteruelas,* Katarzyna A. Mituła-Chmielowiec, Enrique Oñate

Departamento de Química Inorgánica, Instituto de Síntesis Química y Catálisis Homogénea (ISQCH), Centro de Innovación en Química Avanzada (ORFEO-CINQA), Universidad de Zaragoza-CSIC, 50009 Zaragoza, Spain

* Corresponding author's e-mail address: maester@unizar.

CONTENTS

Experimental Section **S2**

Structural Analysis of Complexes 2 and 7 **S4**

Computational Details. **S5**

NMR spectra **S8**

References **S28**

Experimental Section

General information:

All reactions were performed with rigorous exclusion of air using standard Schlenk-tube or glovebox techniques. Solvents were dried by the usual procedures and distilled under argon before use or obtained oxygen- and water-free from an MBraun solvent purification apparatus. Toluene-d_8 and benzene-d_6 were stored over sodium. Complex $OsH_6(P^iPr_3)_2$ was prepared according to the published methods.[1] Alkynes were purchased from commercial sources and distilled in a Kugelrohr distillation oven. NMR spectra were recorded on Bruker Avance 300 MHz instruments. Chemical shifts (expressed in parts per million) are referenced to residual solvent peaks (^1H, $^{13}C\{^1H\}$) and external H_3PO_4 ($^{31}P\{^1H\}$) or $CFCl_3$ ($^{19}F\{^1H\}$). Coupling constants J and $\Delta\upsilon$ are given in Hertz. High-resolution (HRMS) electrospray mass spectra were acquired using a MicroTOF-Q hybrid quadrupole time-of-flight spectrometer (Bruker Daltonics, Bremen, Germany). C, H, and N analyses were carried out in a PerkinElmer 2400 CHNS/O analyzer. Attenuated total reflection infrared spectra (ATR-IR) of solid samples were run on a Perkin-Elmer Spectrum 100 FT-IR spectrometer.

Optimization of the Catalytic Conditions

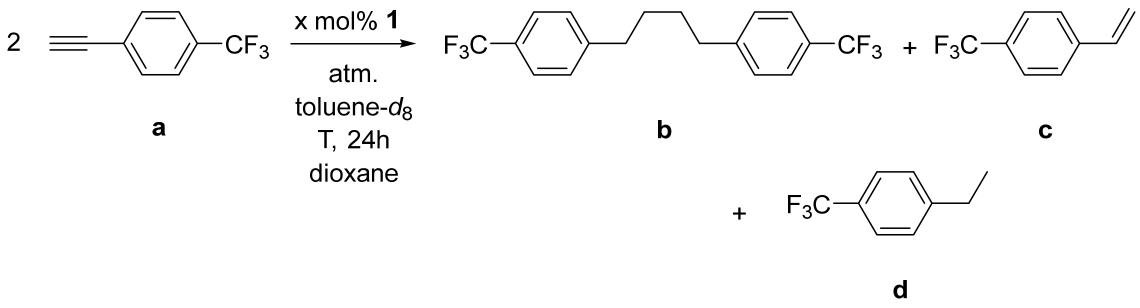

[Os] (mol %)	Tol-d_8 (mL)	atm	T (ºC)	% Yield of a/b/c/d
5	0.5	H_2 (1 atm)	90	0/**25**/0/75
2	0.5	H_2 (1 atm)	90	84/**1**/16/0
7	0.5	H_2 (1 atm)	90	0/**24**/0/76
10	0.5	H_2 (1 atm)	90	0/**22**/0/78
5	0.5	H_2 (1 atm)	50	0/**7**/8/80
5	0.5	H_2 (1 atm)	120	0/**12**/0/88
5	0.5	Ar/H_2 (5% H_2, 1 atm)	90	Polymerization processes
5	0.5	H_2 (2 atm)	90	0/**0**/0/100
5%	-	H_2 (1 atm)	90ºC	Polymerization processes

Structural Analysis of Complexes 2 and 7

X-ray data were collected on a D8 Venture Bruker diffractometer (Mo radiation, λ = 0.71073 Å). The crystals were mounted under oil in a MiTeGen mount and cooled to 100(2) K with an open-flow nitrogen gas (Oxford Cryosystems). Data were corrected for absorption by using a multiscan method applied with the SADABS program.[2] The structures were solved by Patterson or direct methods and refined by full-matrix least squares on F^2 with SHELXL2019,[3] including isotropic and subsequently anisotropic displacement parameters. The hydrogen atoms were observed in the last Fourier Maps or calculated, and refined freely or using a restricted riding model.

The disordered CF_3 groups were refined with different moieties with complementary occupations, restrained geometry, and isotropic displacement parameters.

Crystal data for **3**: $C_{34}H_{58}OsP_2$, M_W 718.94, colorless, irregular block (0.152 x 0.143 x 0.090 mm^3), monoclinic, space group $P2_1/c$, *a*: 11.6501(4) Å, *b*: 16.2445(6) Å, *c*: 17.4687(6) Å, *β*: 95.2633(12)°, *V* = 3292.0(2) Å^3, *Z* = 4, *Z'* = 1, D_{calc}: 1.451 g cm^{-3}, F(000): 1472, T = 100(2) K, μ = 3.991 mm^{-1}. 84080 measured reflections (2θ: 3-57°, ω and φ scans 0.5°), 8188 unique (R_{int} = 0.0351); min./max. transm. factors 0.644/0.746. Final agreement factors were R^1 = 0.0138 (7987 observed reflections, I > 2σ(I)) and wR^2 = 0.0319; data/restraints/parameters 8188/0/364; GoF = 1.039. Largest peak and hole 0.714 (close to Os atoms) and -1.078 e/ Å^3.

Crystal data for **7**: $C_{36}H_{52}F_6OsP_2$, 0.5(C_5H_{12}), M_W 886.99, yellow, irregular block, (0.162 x 0.075 x 0.043 mm^3), triclinic, space group P-1, *a*: 15.8286(8) Å, *b*: 17.6928(9) Å, *c*: 17.8370(10) Å, *a*: 112.6592(19)°, *β*: 91.064(2)°, *γ*: 115.6711(19)°, *V* = 4050.1(4) Å^3, *Z* = 4, *Z'* = 2, D_{calc}: 1.455 g cm^{-3}, F(000): 1796, T = 100(2) K, μ = 3.280 mm^{-1}. 273288 measured reflections (2θ: 3-57°, ω and φ scans 0.5°), 20143 unique (R_{int} = 0.0395); min./max. transm. factors 0.645/0.746. Final agreement factors were R^1 = 0.0361 (18682 observed reflections, I > 2σ(I)) and wR^2 = 0.0867; data/restraints/parameters 20143/31/ 926; GoF = 1.082. Largest peak and hole 5.411 (close to Os atoms) and -2.571 e/ Å^3.

Computational Details.

All calculations were performed at the DFT level using the B3LYP functional[4] supplemented with Grimme's dispersion correction D3[5] as implemented in Gaussian09.[6] Os atoms were described by means of an effective core potential SDD for the inner electron[7] and its associated double-ζ basis set for the outer ones, complemented with a set of f-polarization functions for osmium.[8] The 6-31G** basis set was used for all the other atoms.[9] The SMD continuum model was used to model the effects of the solvent (toluene) in the reactivity studies. Reactants, intermediates, and products were also characterized by frequency calculations and have positive definite Hessian matrices thus confirming that the computed structure is a minimum on the potential energy surface. Transition states were identified by having one imaginary frequency in the Hessian matrix. It was confirmed that transition states connect with the corresponding intermediates by means of the application of an eigenvector corresponding to the imaginary frequency and subsequent optimization of the resulting structures. Gibbs energies were computed at 298.15 K and 1 atmosphere. All values collected in the figures correspond to Gibbs energies in kcal mol^{-1}.

Energies of Optimized Structures

**Compound**	**Sum of electronic and thermal Free Energies (Hartree/Particle)**
**A**	-1486.727420
**F**	-1795.069407
**TS(F-G)**	-1795.065388
**G**	-1795.079421
**TS(G-B)**	-1795.075877
**B**	-1795.089046
**TS(B-C)**	-1795.078285
**C**	-1795.112824
**I**	-2103.450259
**TS(I-D)**	-2103.442919
**D**	-2103.455237
**TS(D-E)**	-2103.448030
**E**	-2103.480854
**TS(E-2)**	-2103.454193
**2**	-2103.531276
**TS(D-^{1}D)**	-2103.420307
**^{1}D**	-2103.487902
**TS(^{1}D-2)**	-2103.472142
**H_2**	-1.179365
**PhC≡CH**	-308.340121

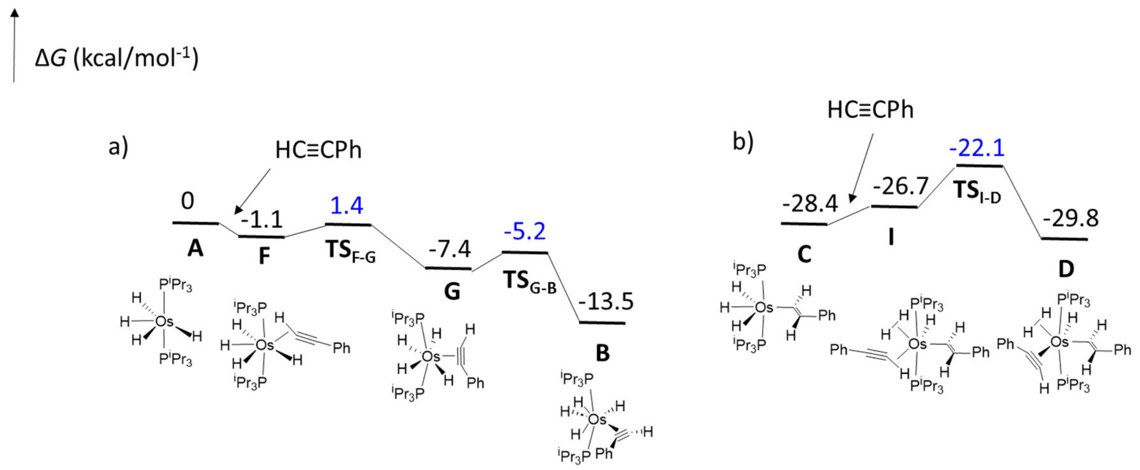

Figure S1: Coordination and σ-π slippage of phenylacetylene over **A** and **C** intermediates. The Cartesian coordinates of the optimized intermediates and transition states can be found in the xyz supplementary file and visualized with free software like Mercury.

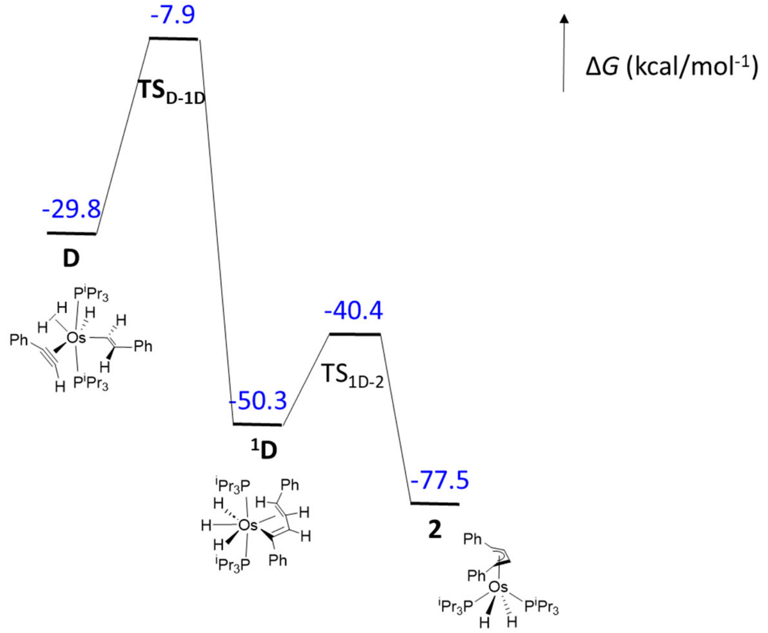

Figure S2: Alternative pathway for the C-C coupling over the intermediate **D**. The Cartesian coordinates of the optimized intermediates and transition states can be found in the xyz supplementary file and visualized with free software like Mercury.

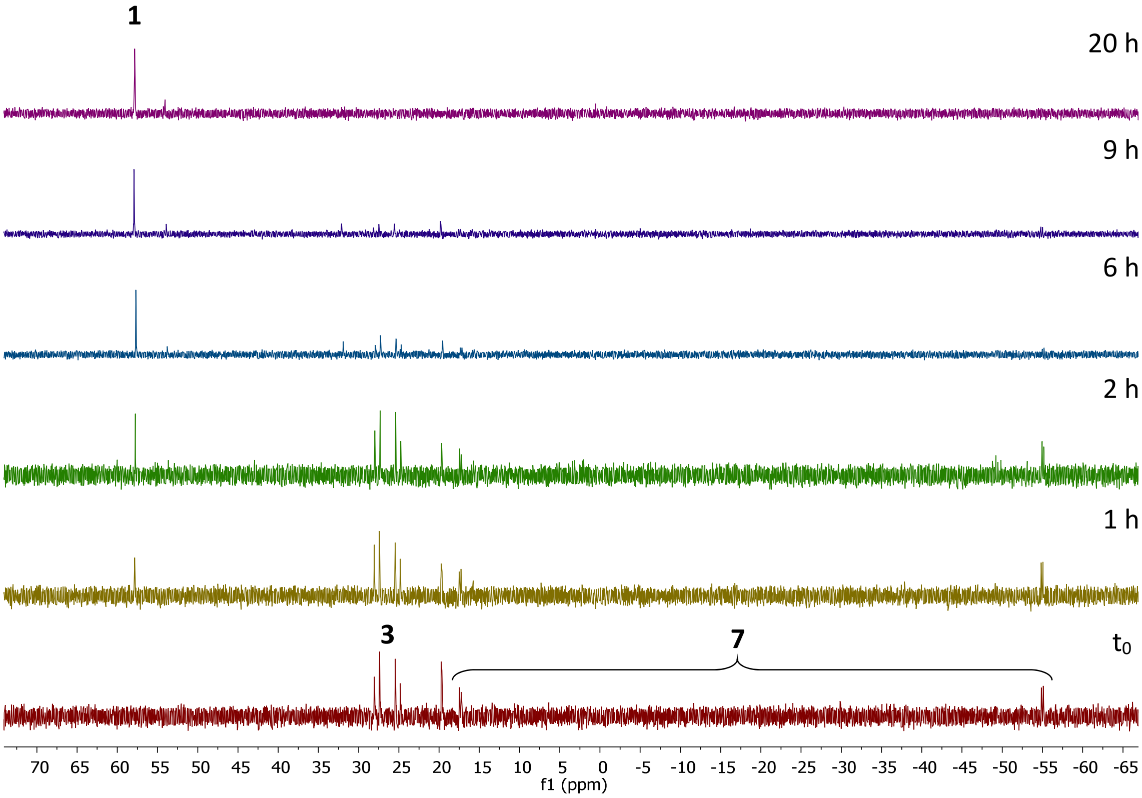

Figure S3. Stacked $^{31}P\{^{1}H\}$ NMR spectra showing the evolution of **1** from the mixture of complexes **3** and **7** under 1 atm of H_2 at 90 ºC by the time.

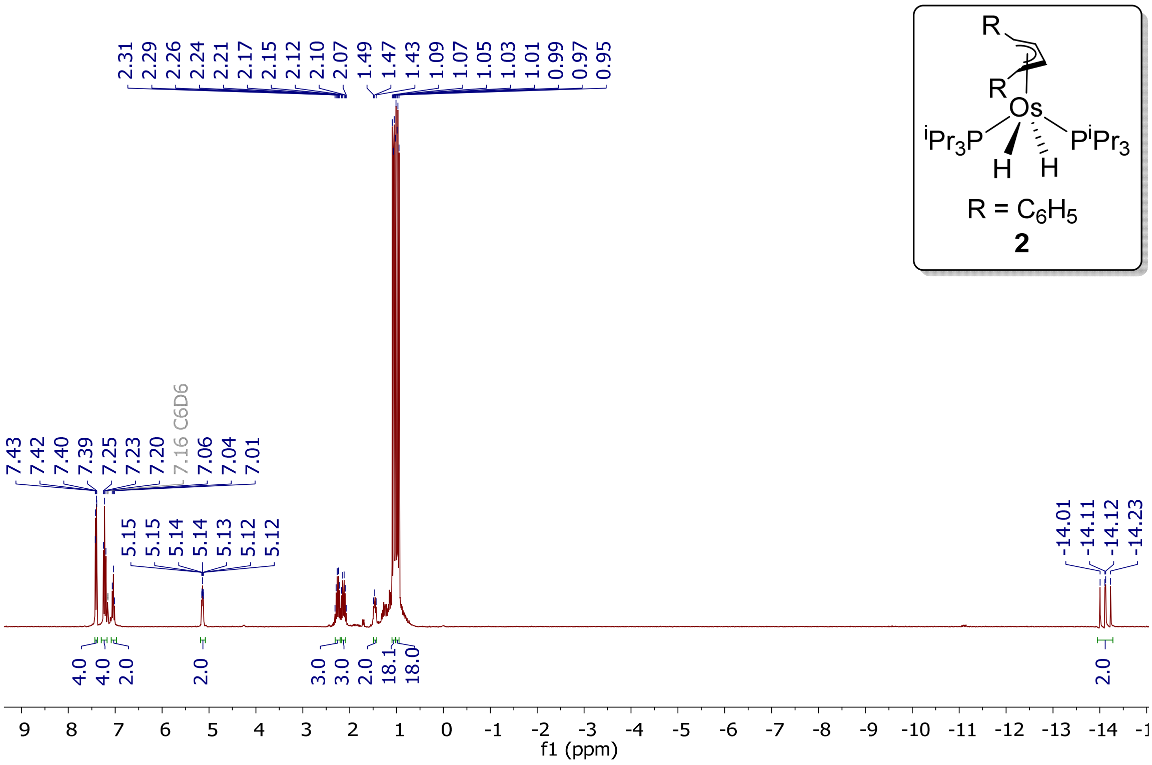

Figure S4. ^{1}H NMR spectrum (300.13 MHz, C_6D_6, 298 K) of complex **2**.

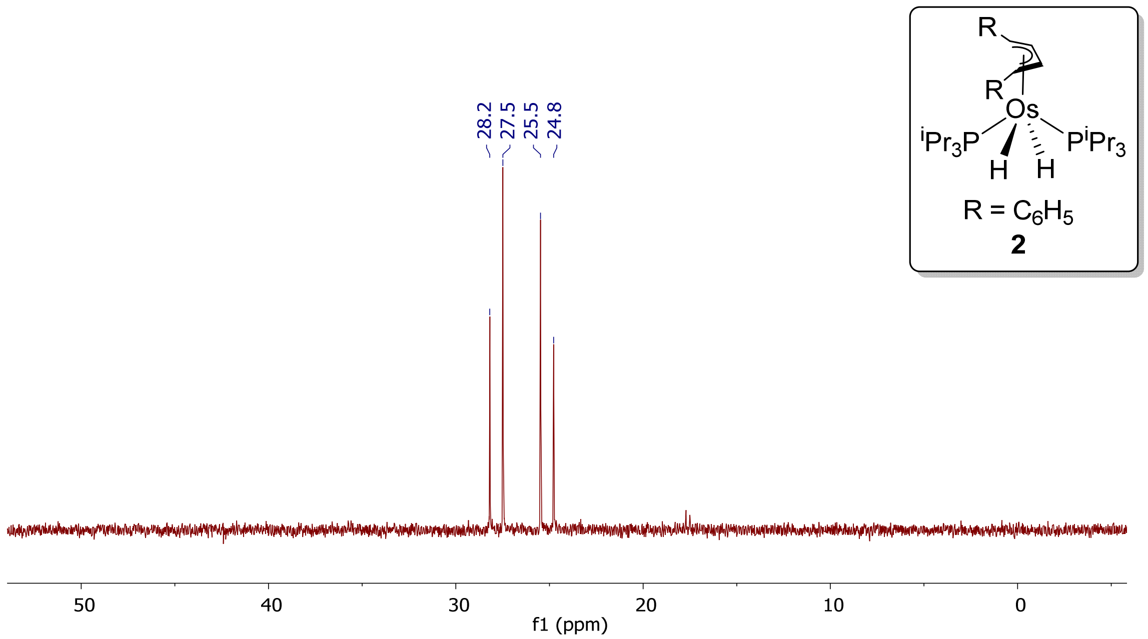

Figure S5. $^{31}P\{^{1}H\}$ NMR spectrum (121.50 MHz, C_6D_6, 298 K) of complex **2**.

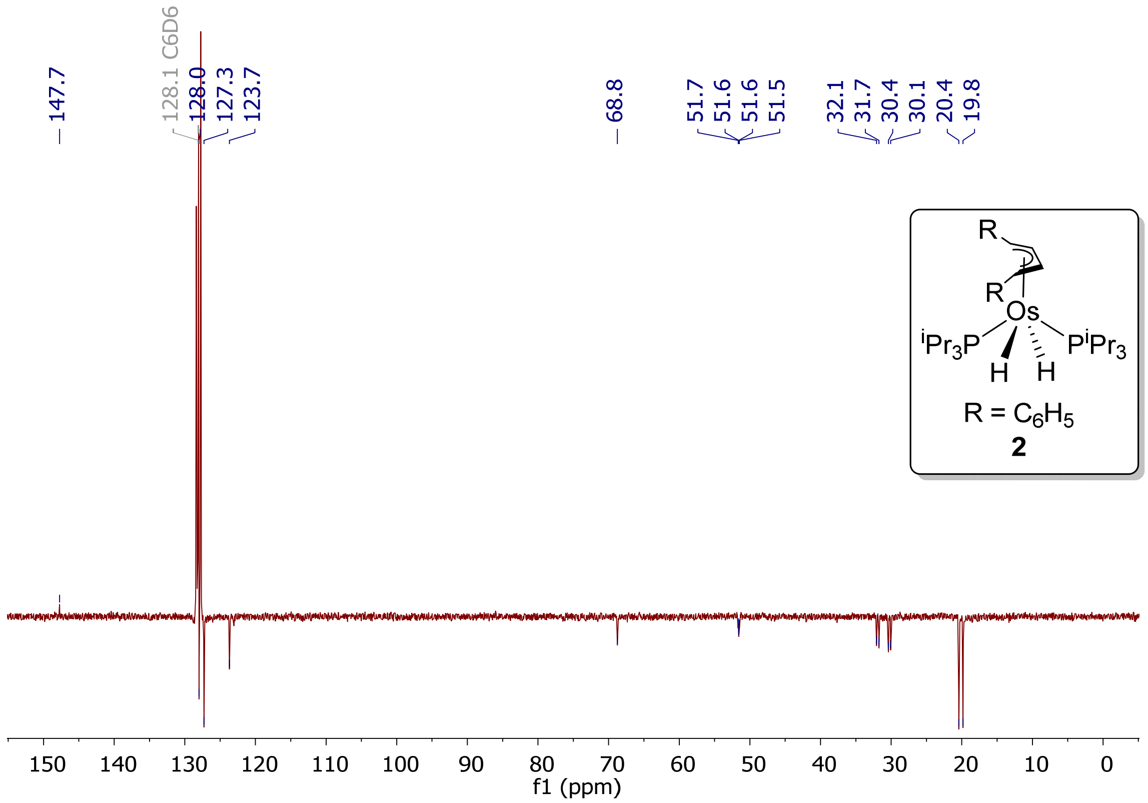

Figure S6. $^{13}C\{^{1}H\}$-APT NMR spectrum (75.48 MHz, C_6D_6, 298 K) of complex **2**.

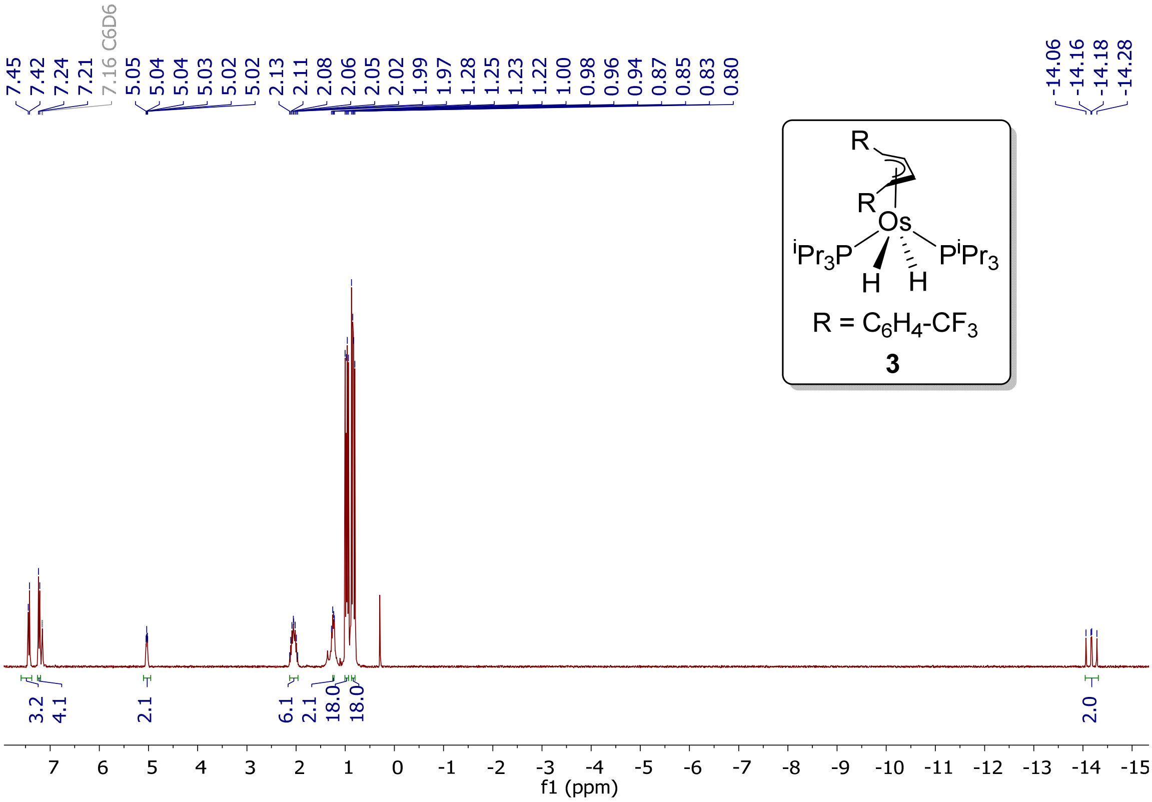

Figure S7. ^{1}H NMR spectrum (300.13 MHz, C_6D_6, 298 K) of complex **3**.

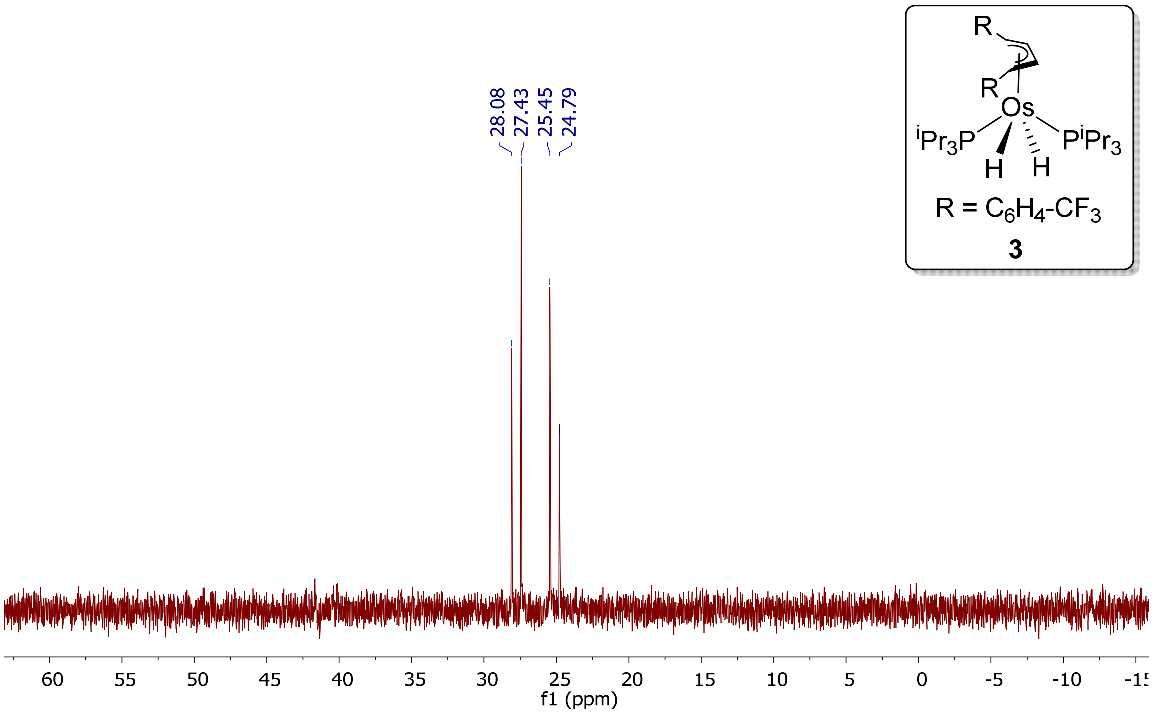

Figure S8. $^{31}P\{^{1}H\}$ NMR spectrum (121.50 MHz, C7D8, 298 K) of complex **3**.

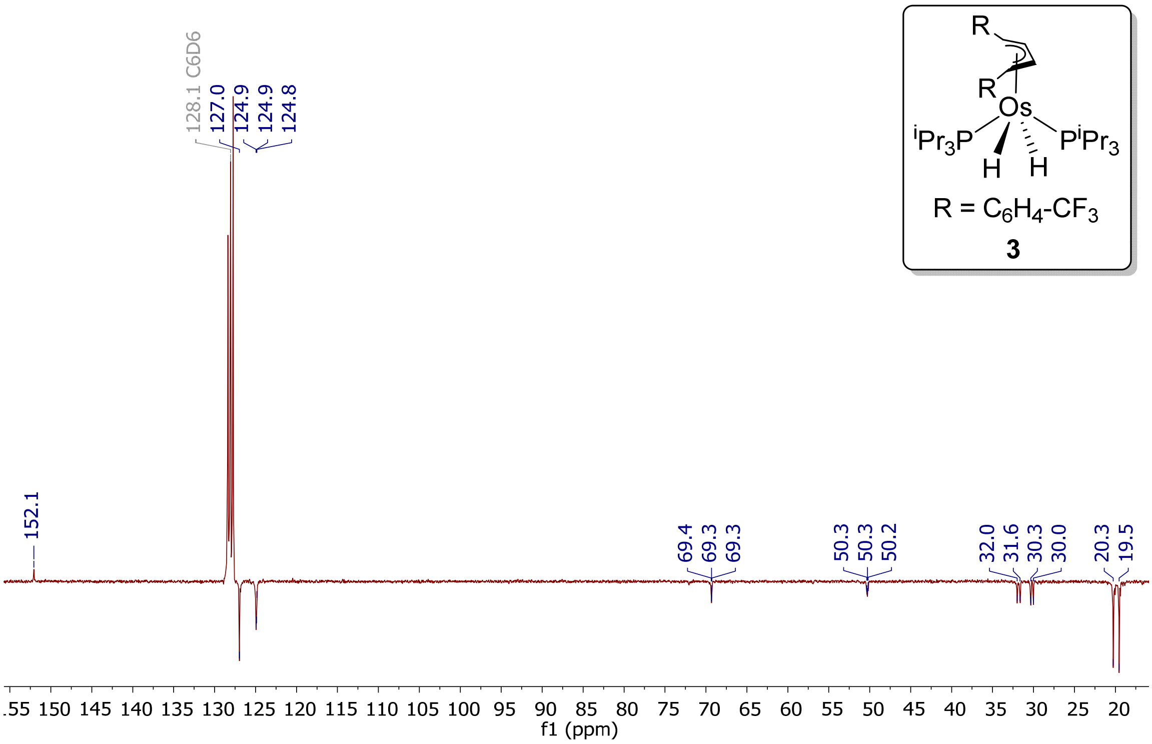

Figure S9. $^{13}C\{^{1}H\}$-APT NMR spectrum (75.48 MHz, C_6D_6, 298 K) of complex **3**.

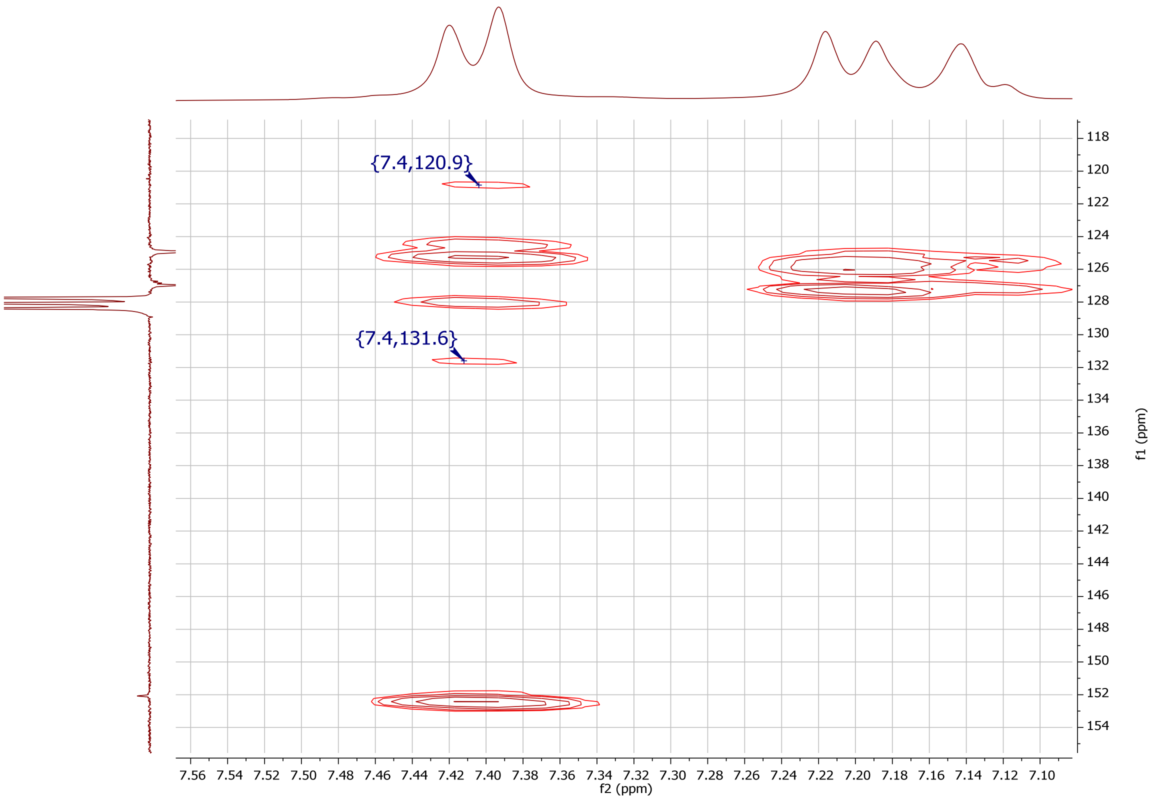

Figure S10. HMBC $\{^{1}H, ^{13}C\}$ NMR spectrum (300.13, 75.48 MHz, C_6D_6, 298 K) of complex **3**.

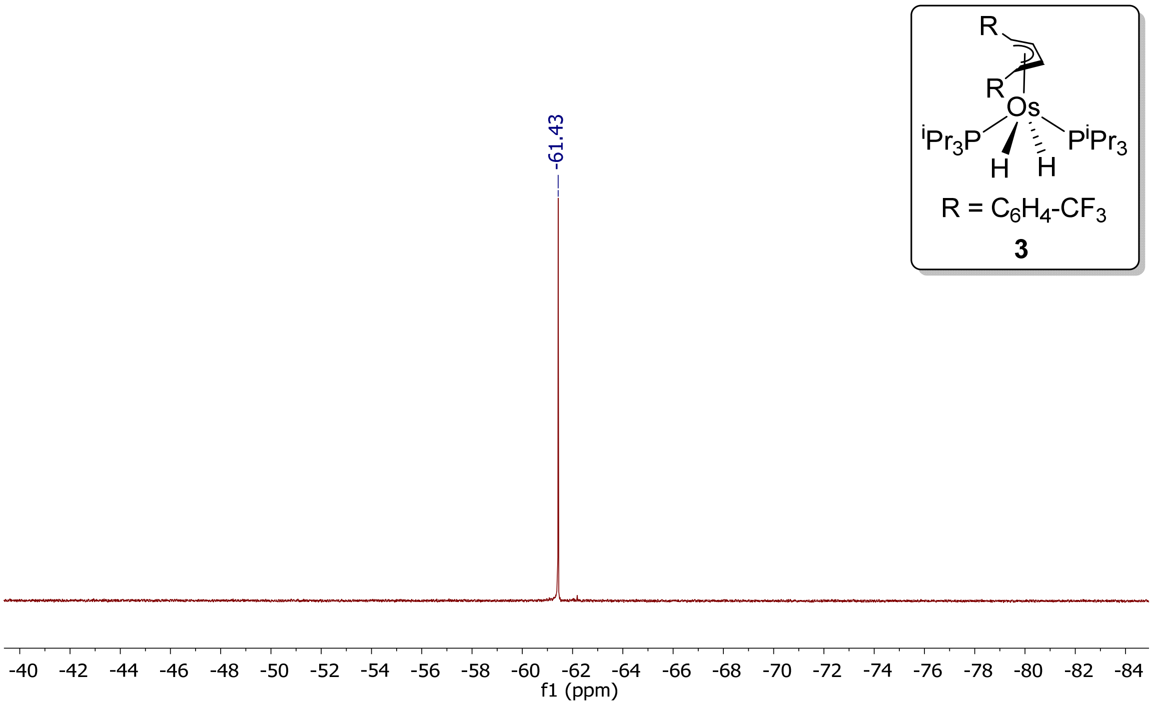

Figure S11. ^{11}F{^{1}H} NMR spectrum (282.38 MHz, C_6D_6, 298 K) of complex **3**.

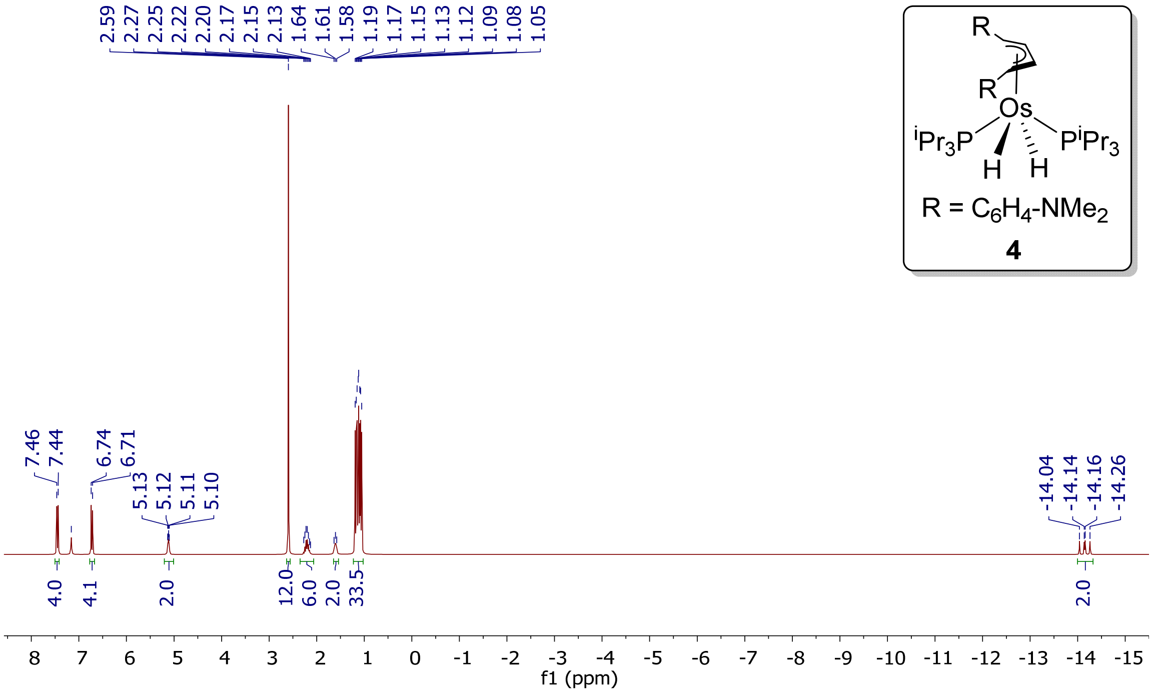

Figure S12. ^{1}H NMR spectrum (300.13 MHz, C_6D_6, 298 K) of complex **4**.

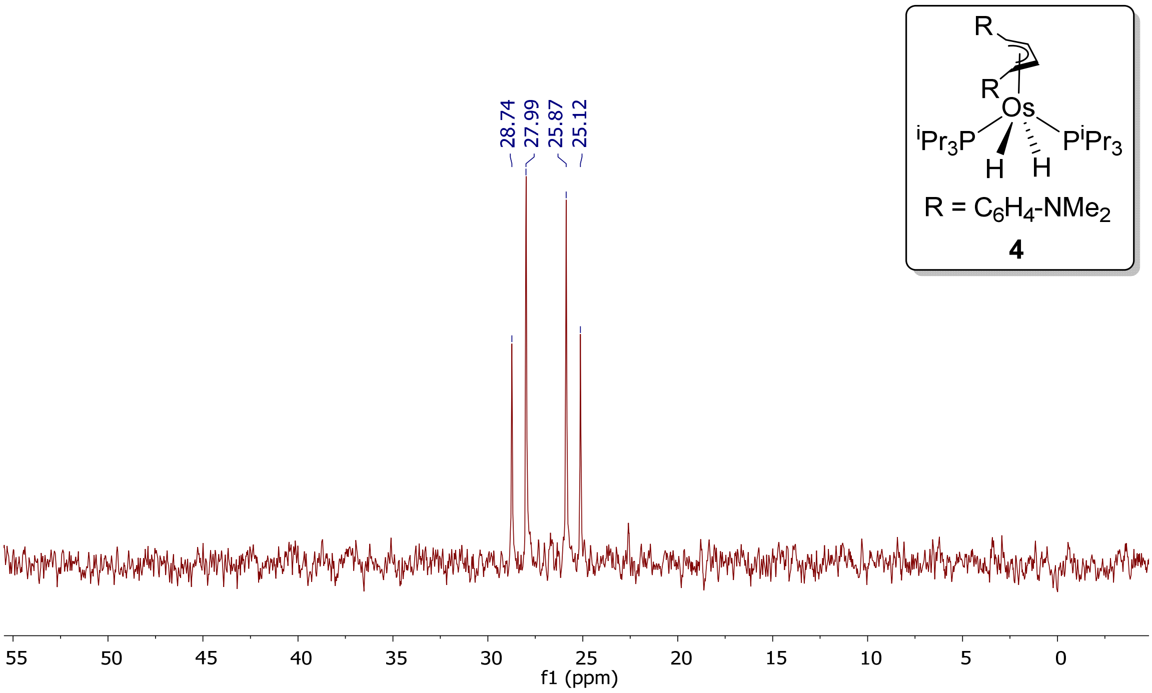

Figure S13. $^{31}P\{^{1}H\}$ NMR spectrum (121.50 MHz, C_6D_6, 298 K) of complex **4**.

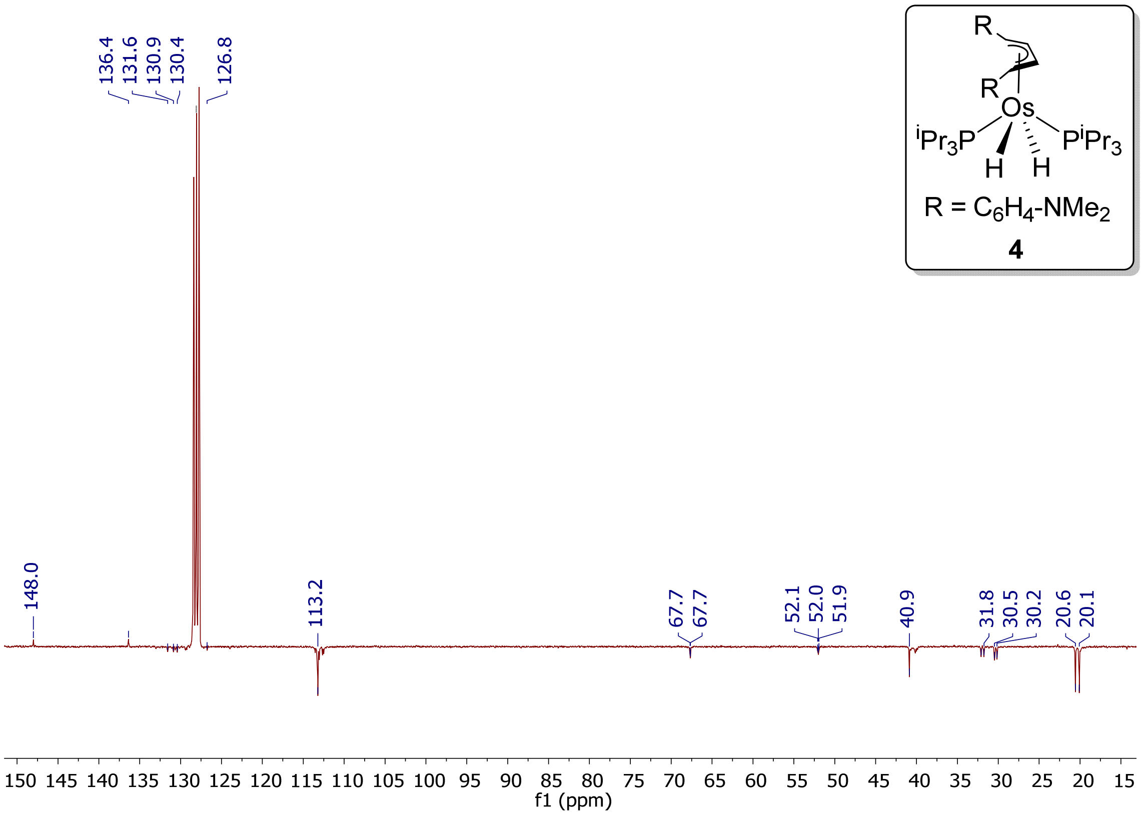

Figure S14. $^{13}C\{^{1}H\}$-APT NMR spectrum (75.48 MHz, C_6D_6, 298 K) of complex **4**.

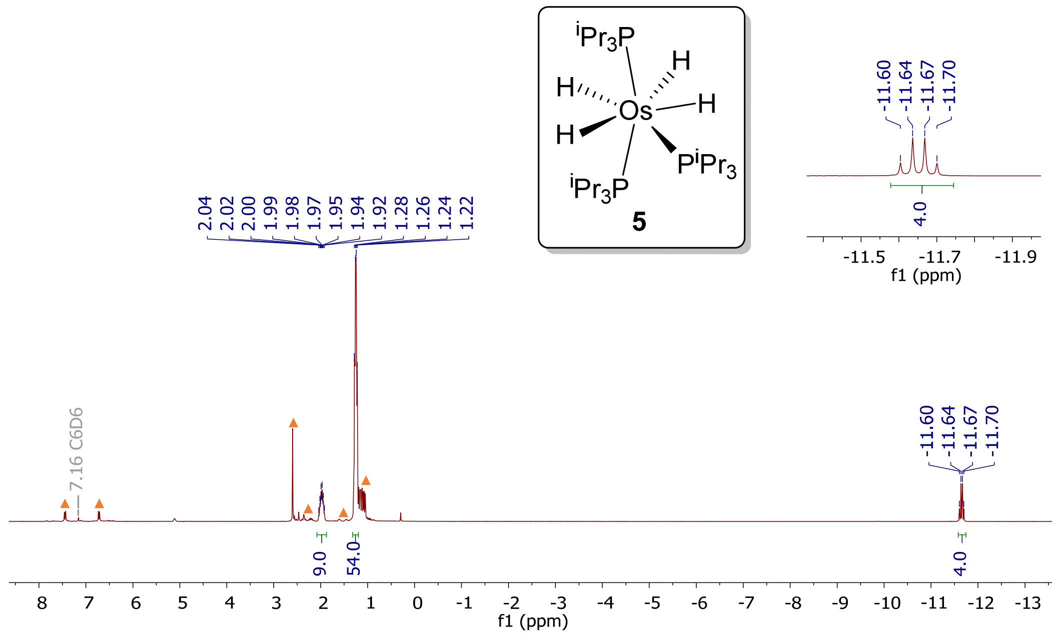

Figure S15. ^{1}H NMR spectrum (300.13 MHz, C_6D_6, 298 K) of complex **5**. Orange triangles denote impurities of complex **4**.

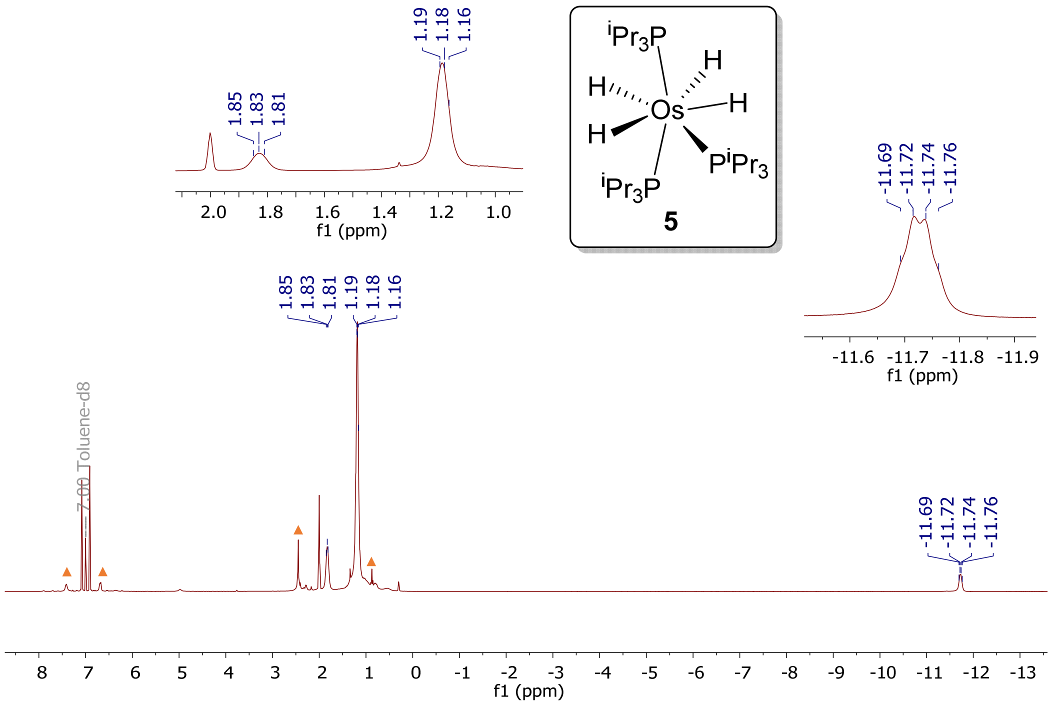

Figure S16. ^{1}H NMR spectrum (400.13 MHz, C_7D_8, 183 K) of complex **5**. Orange triangles denote impurities of complex **4**.

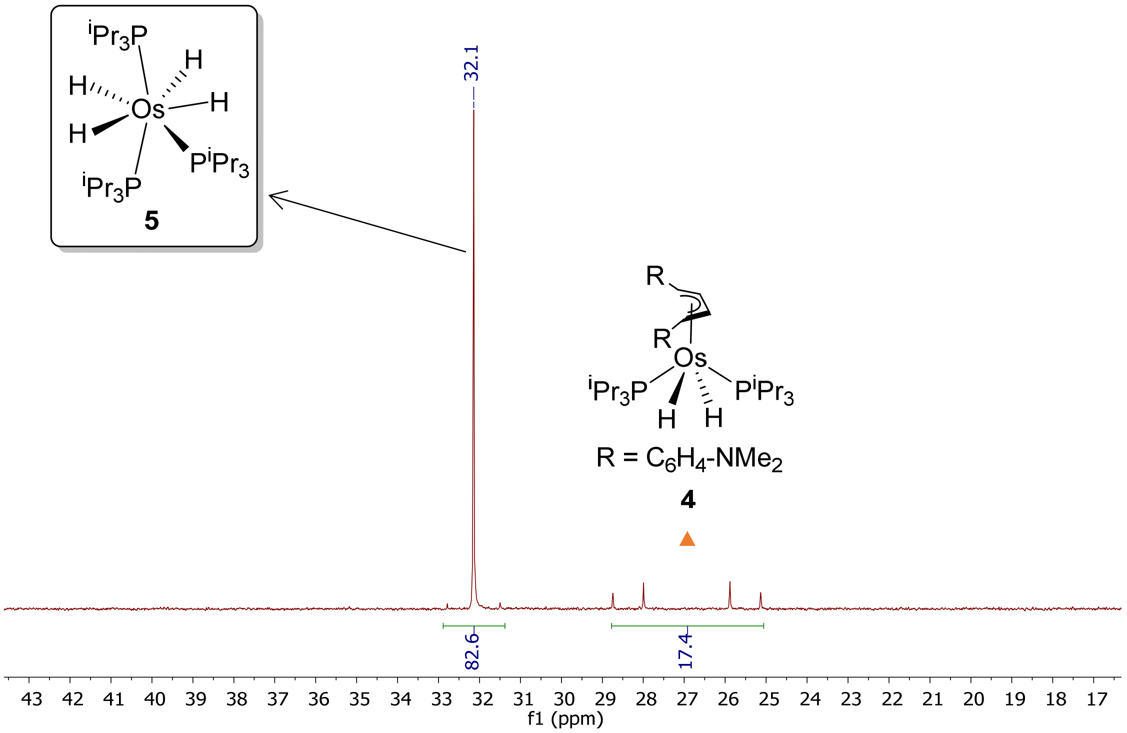

Figure S17. $^{31}P\{^{1}H\}$ NMR spectrum (121.50 MHz, C_6D_6, 298 K) of complex **5**. Orange triangle denotes impurities of complex **4**.

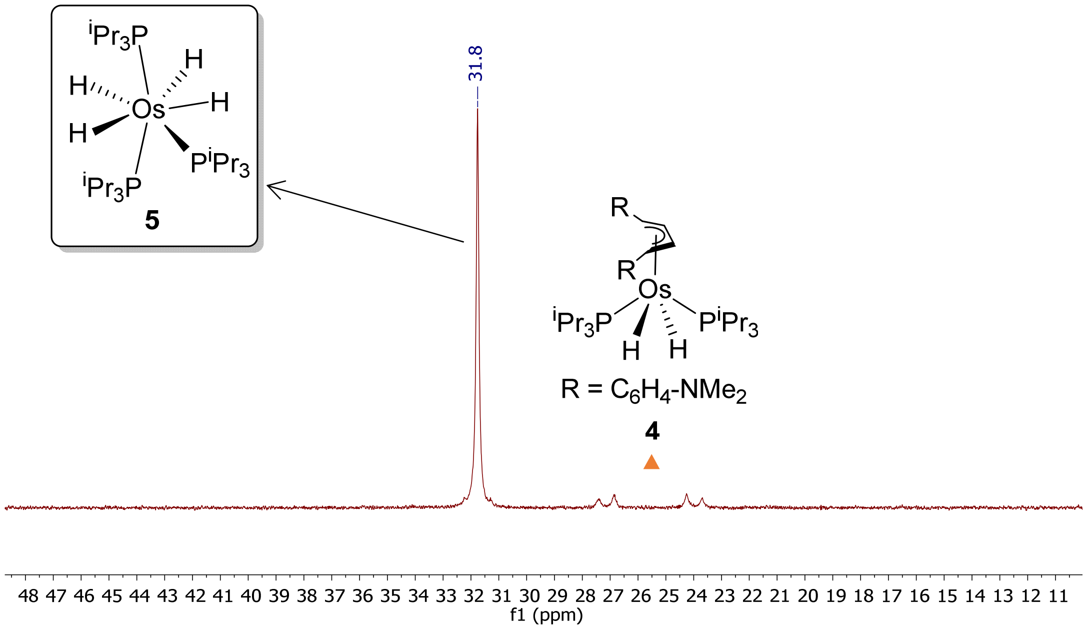

Figure S18. $^{31}P\{^{1}H\}$ NMR spectrum (161.98 MHz, C_7D_8, 183 K) of complex **5**. Orange triangle denotes impurities of complex **4**.

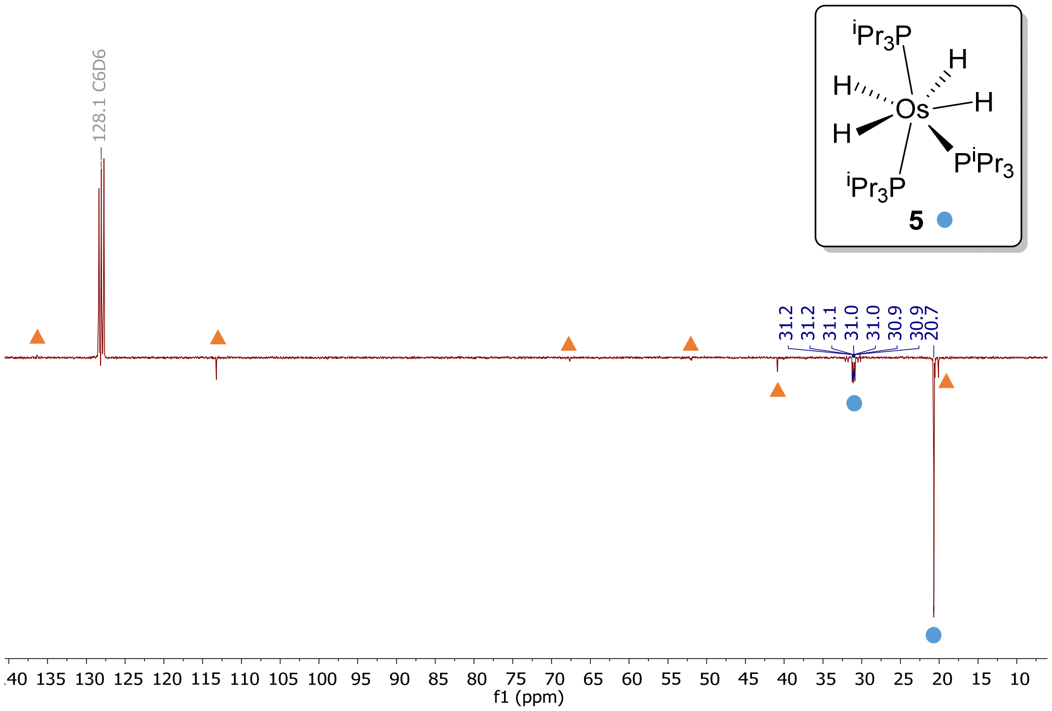

Figure S19. $^{13}C\{^{1}H\}$-APT NMR spectrum (75.48 MHz, C_6D_6, 298 K) of complex **5** (blue spots). Orange triangles denote impurities of complex **4**.

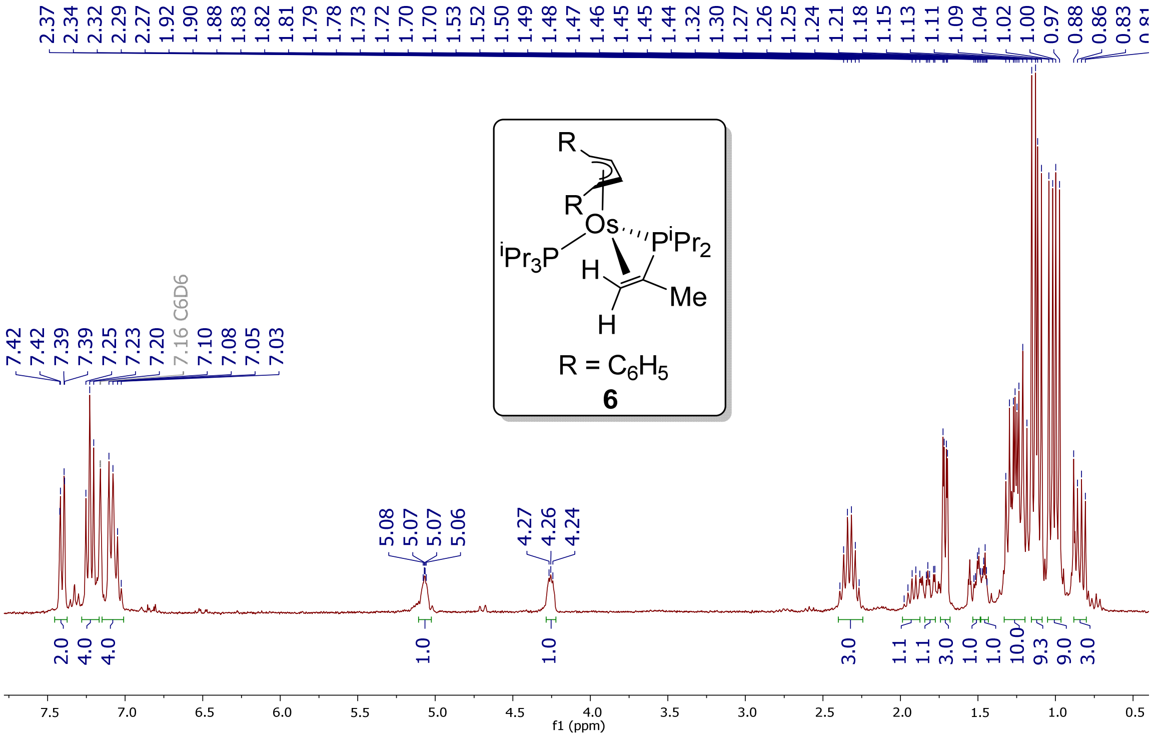

Figure S20. ^{1}H NMR spectrum (300.13 MHz, C_6D_6, 298 K) of complex **6**.

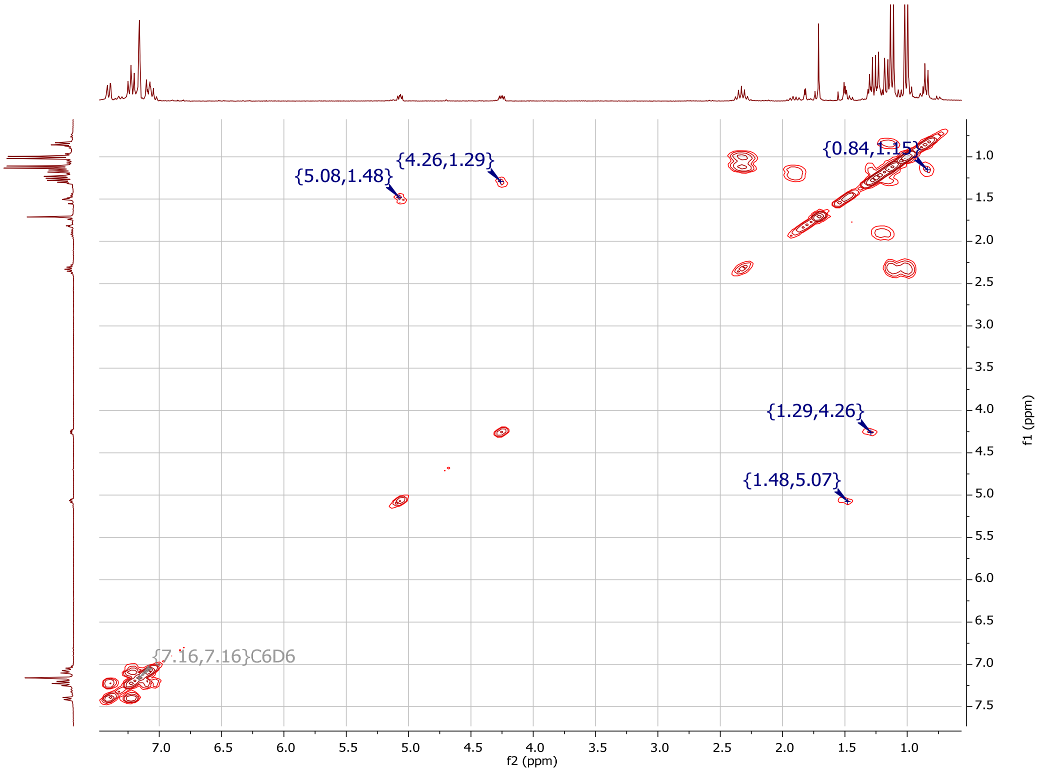

Figure S21. COSY {^{1}H, ^{1}H} NMR spectrum (300.13 MHz, C_6D_6, 298 K) of complex **6**.

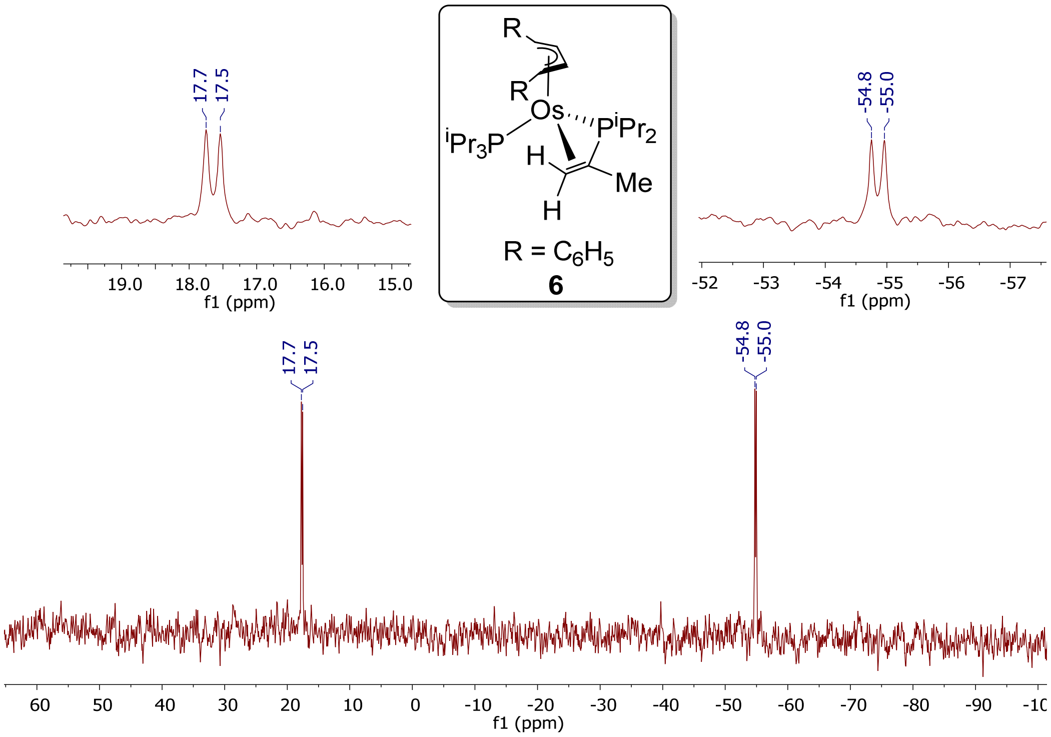

Figure S22. ^{31}P{^{1}H} NMR spectrum (121.50 MHz, C_6D_6, 298 K) of complex **6**.

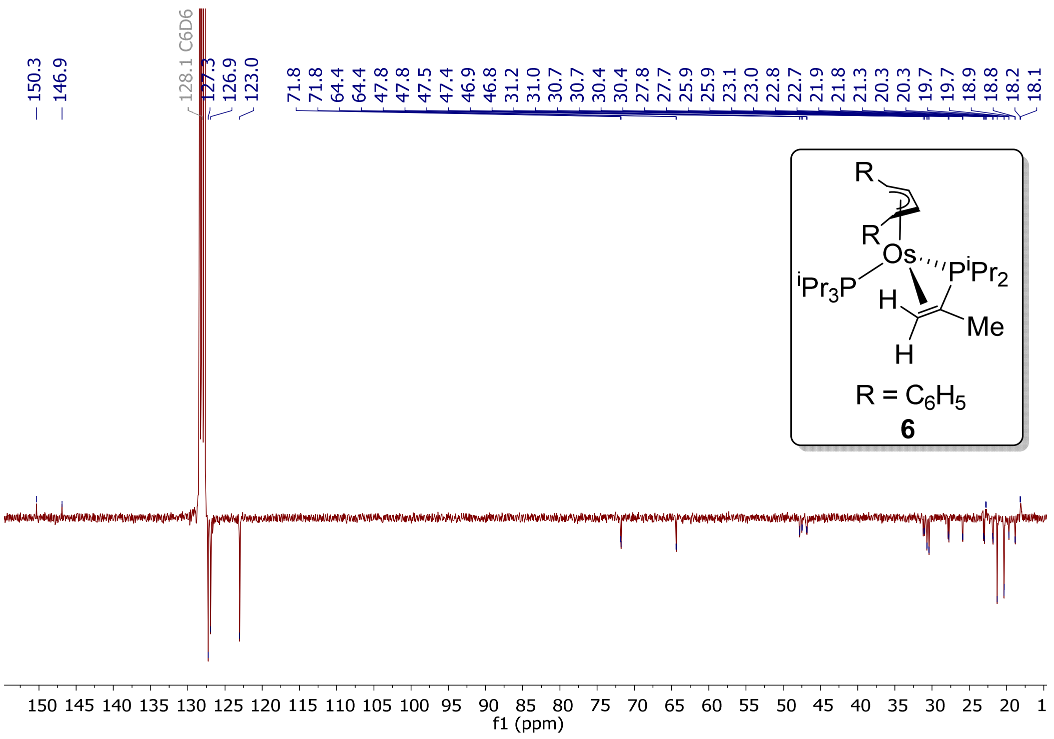

Figure S23. $^{13}C\{^{1}H\}$-APT NMR spectrum (75.48 MHz, C_6D_6, 298 K) of complex **6**.

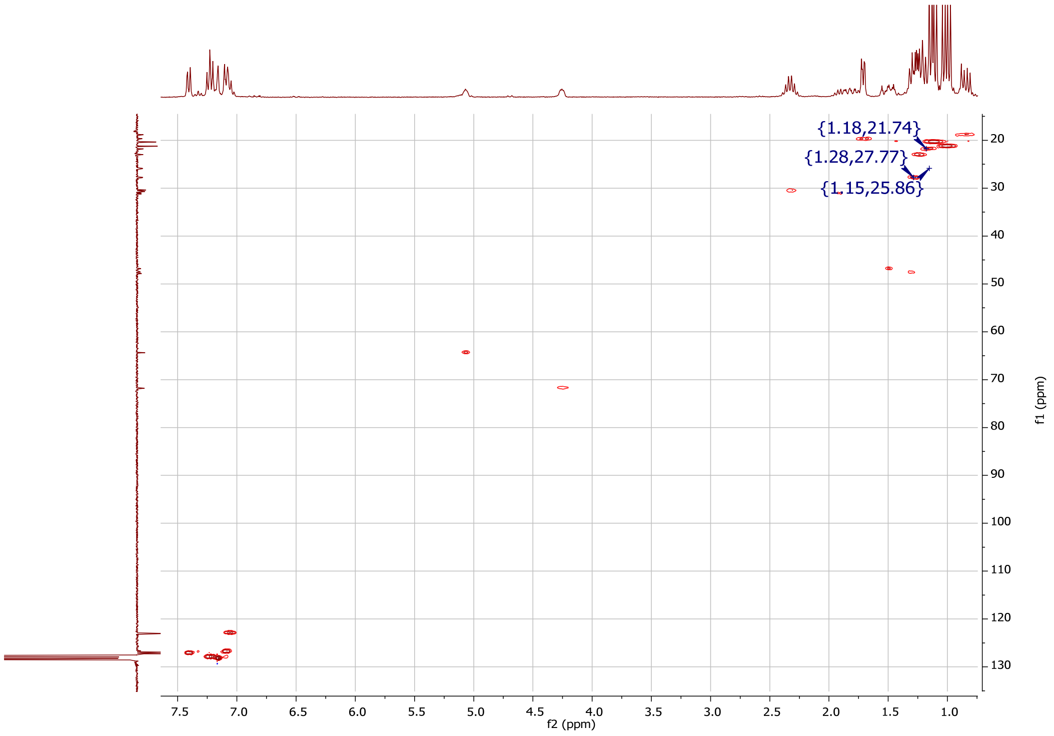

Figure S24. HSQC$\{^{1}H, ^{13}C\}$ NMR spectrum (300.13, 75.48 MHz, C_6D_6, 298 K) of complex **6**.

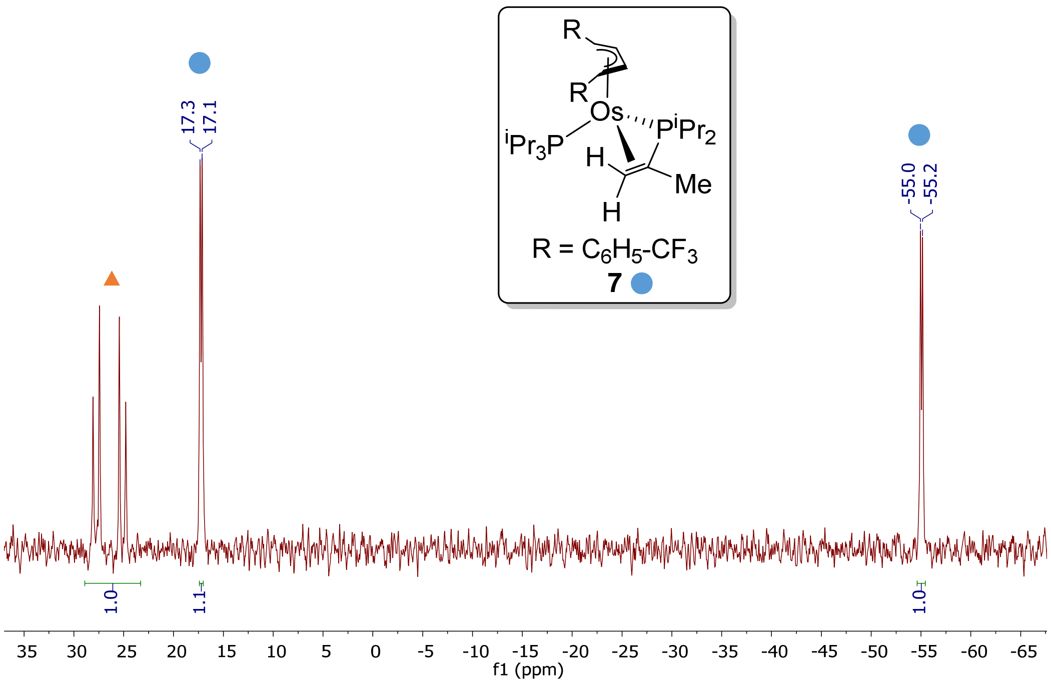

Figure S25. $^{31}P\{^{1}H\}$ NMR spectrum (121.50 MHz, C_6D_6, 298 K) of the mixture of complexes **7** (blue spots) and **3** (orange triangle).

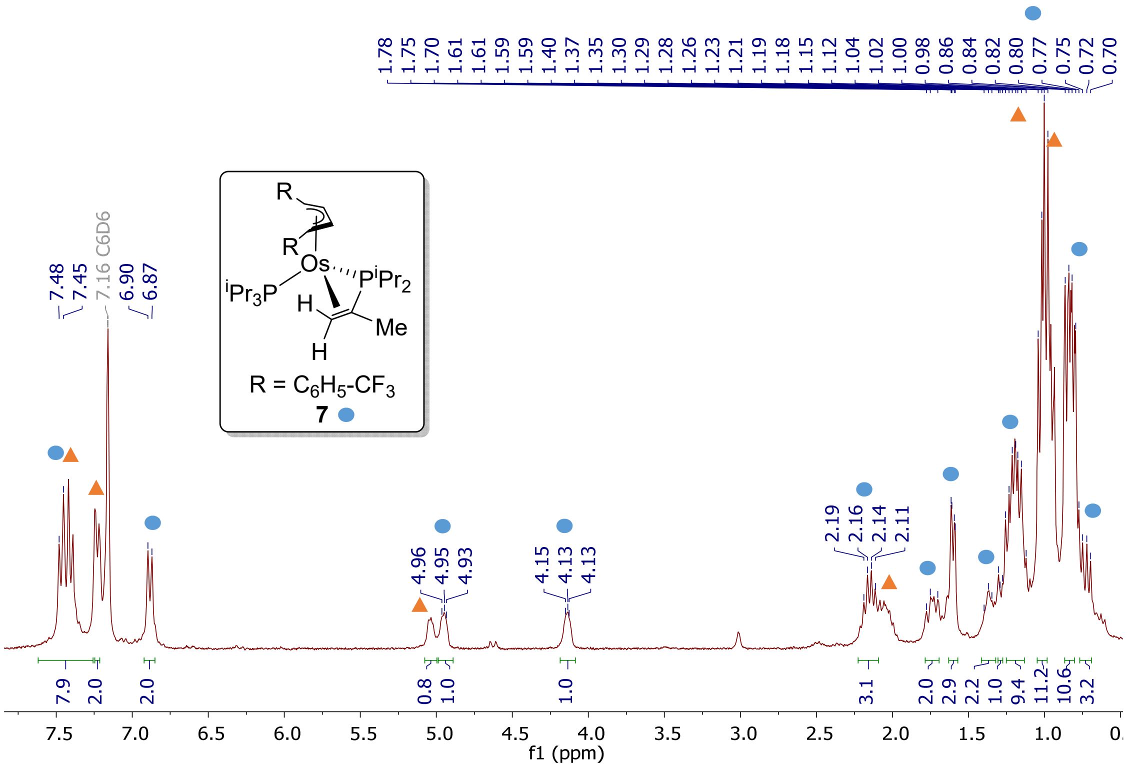

Figure S26. ^{1}H NMR spectrum (300.13 MHz, C_6D_6, 298 K) of the mixture of complexes **7** (blue spots) and **3** (orange triangles).

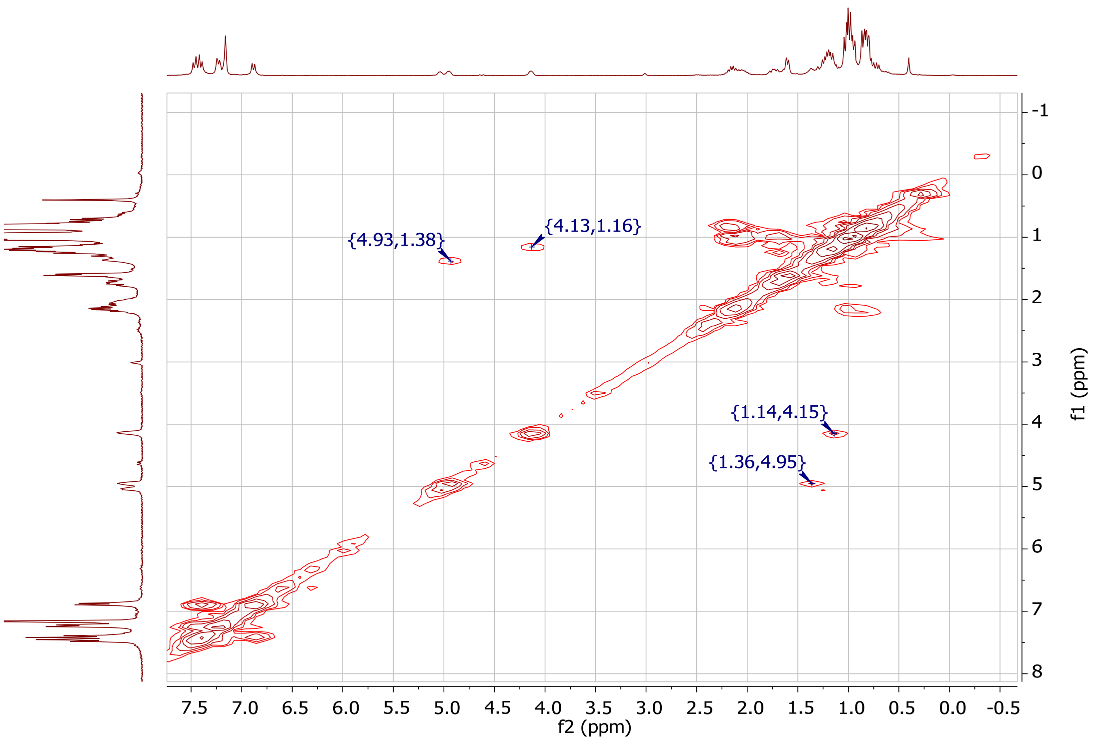

Figure S27. COSY {^{1}H, ^{1}H} NMR spectrum (300.13 MHz, C_6D_6, 298 K) of the mixture of complexes **7** and **3**. Signals corresponding to the C_4H_4 of the butenyl ligand of complex **7** are marked.

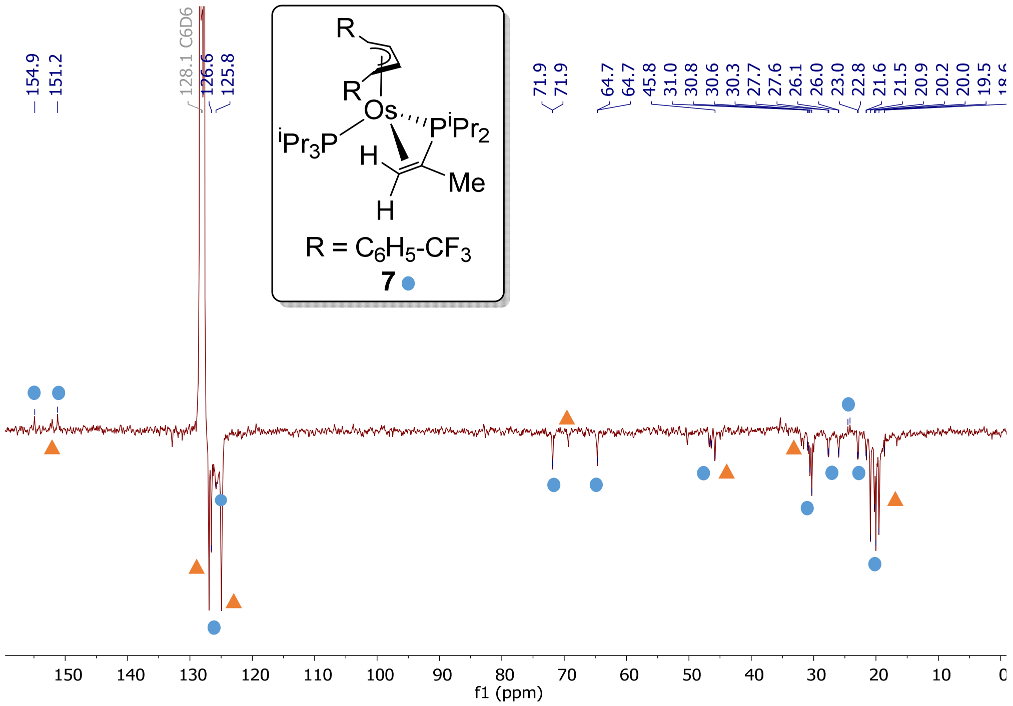

Figure S28. ^{13}C{^{1}H}-APT NMR spectrum (75.48 MHz, C_6D_6, 298 K) of the mixture of complexes **7** (blue spots) and **3** (orange triangles).

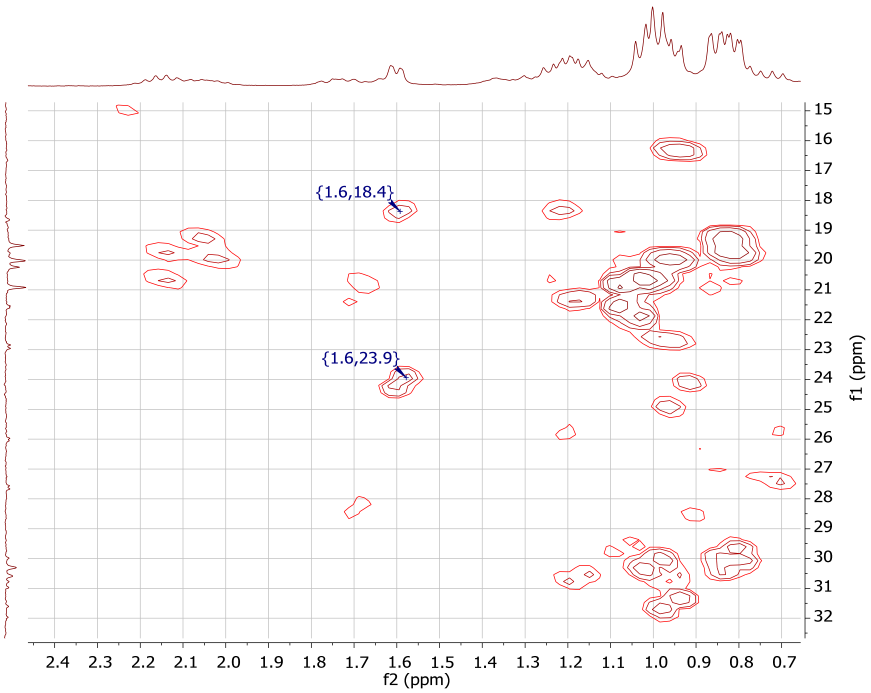

Figure S29. HMBC {^{1}H, ^{13}C} NMR spectrum (300.13, 75.48 MHz, C_6D_6, 298 K) of the mixture of complexes **3** and **7**. Signals corresponding to P(CH_3)*C*=*C*H_2 are marked.

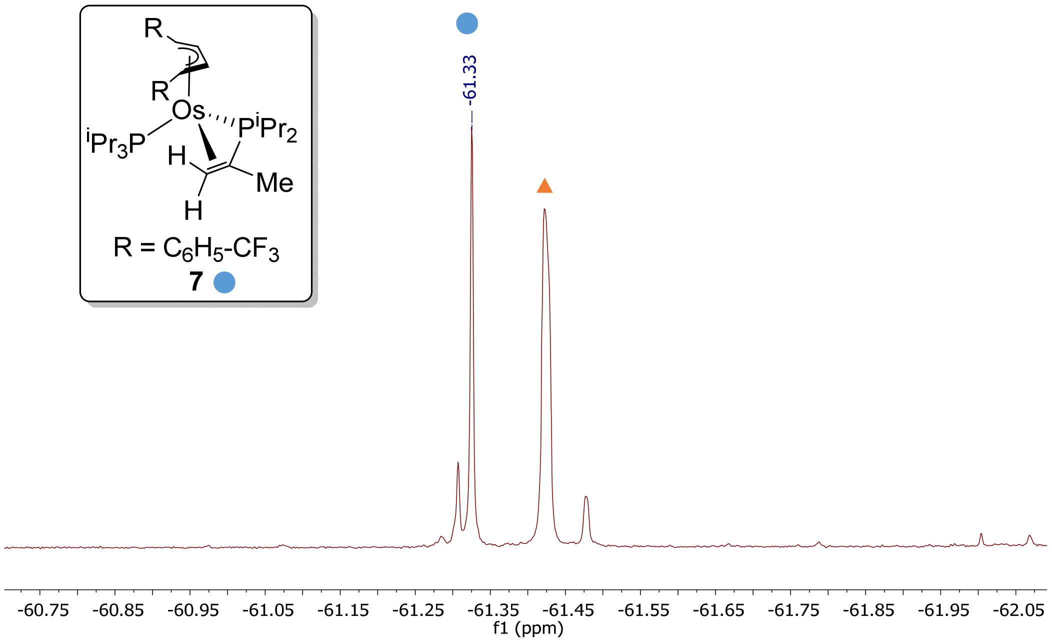

Figure S30. ^{19}F{^{1}H} NMR spectrum (282.38 MHz, C_6D_6, 298 K) of the mixture of complexes **7** (blue spots) and **3** (orange triangles).

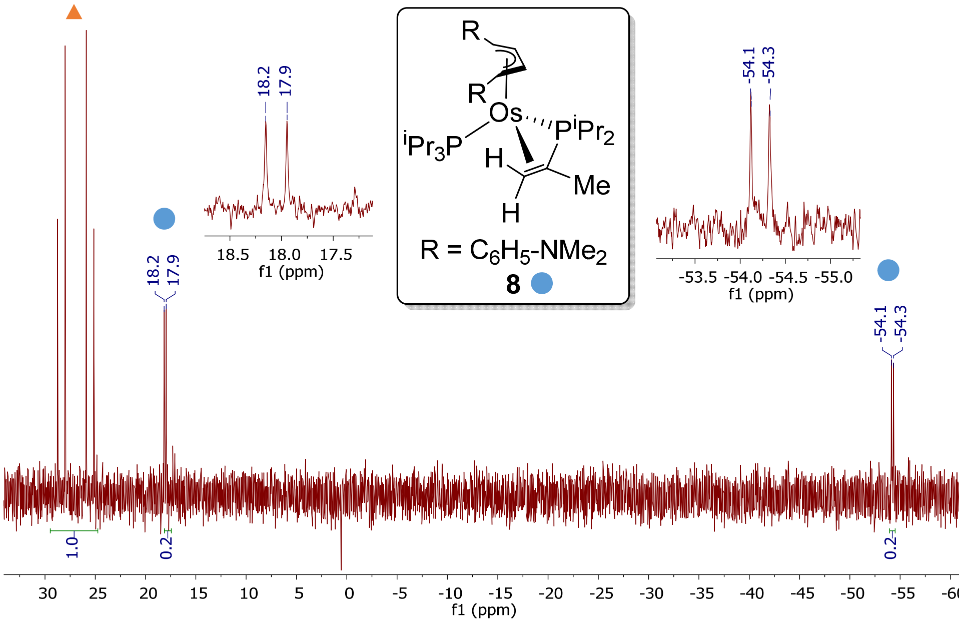

Figure S31. $^{31}P\{^1H\}$ NMR spectrum (121.50 MHz, C_6D_6, 298 K) of the mixture of complexes **8** (blue spots) and **4** (orange triangle).

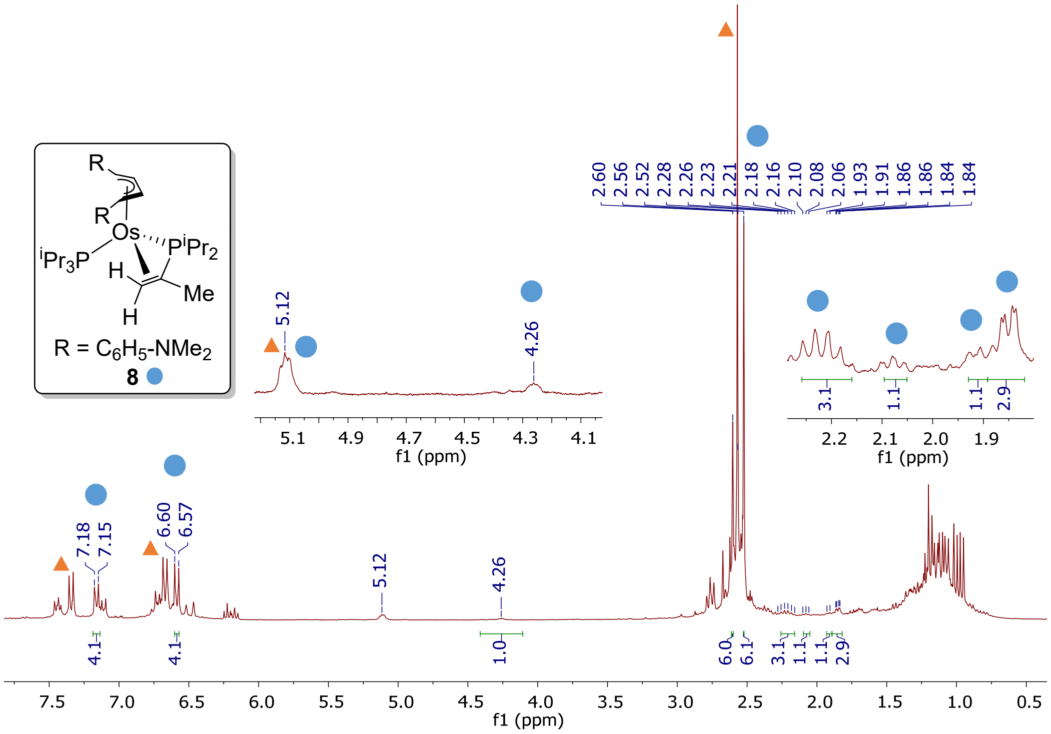

Figure S32. ^1H NMR spectrum (300.13 MHz, C_6D_6, 298 K) of the mixture of complexes **8** (blue spots) and **4** (orange triangle).

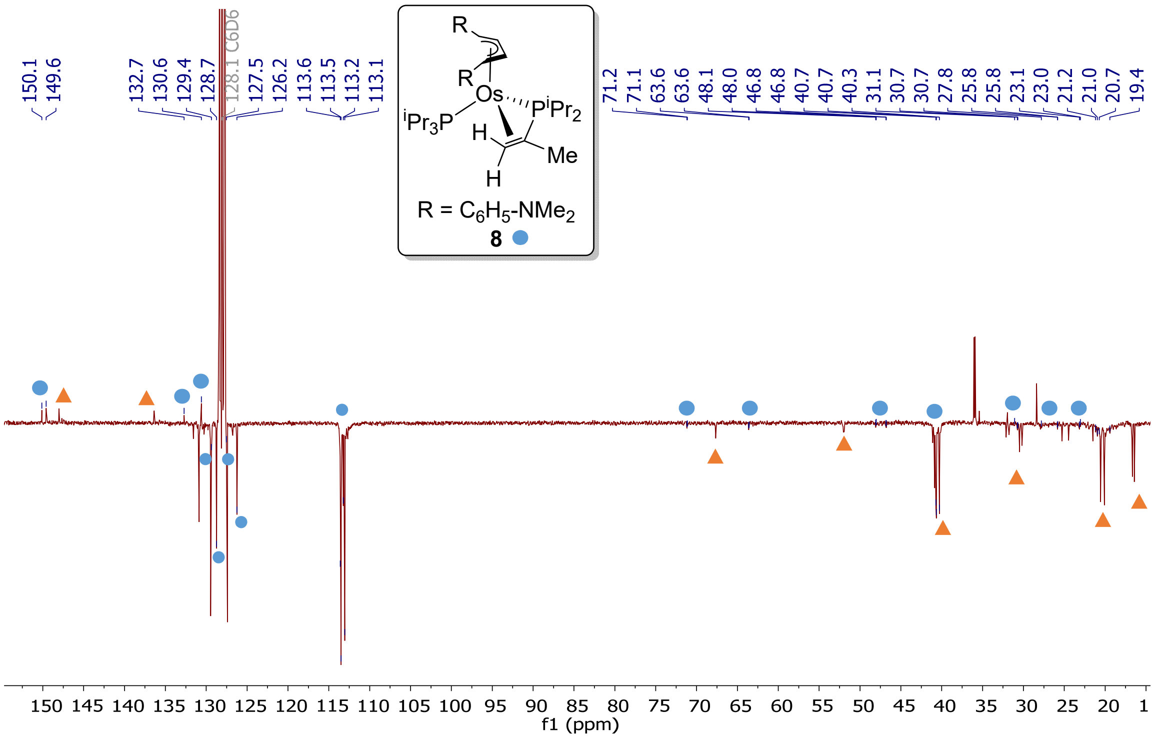

Figure S33. $^{13}C\{^1H\}$-APT NMR spectrum (75.48 MHz, C_6D_6, 298 K) of the mixture of complexes **8** (blue spots) and **4** (orange triangle).

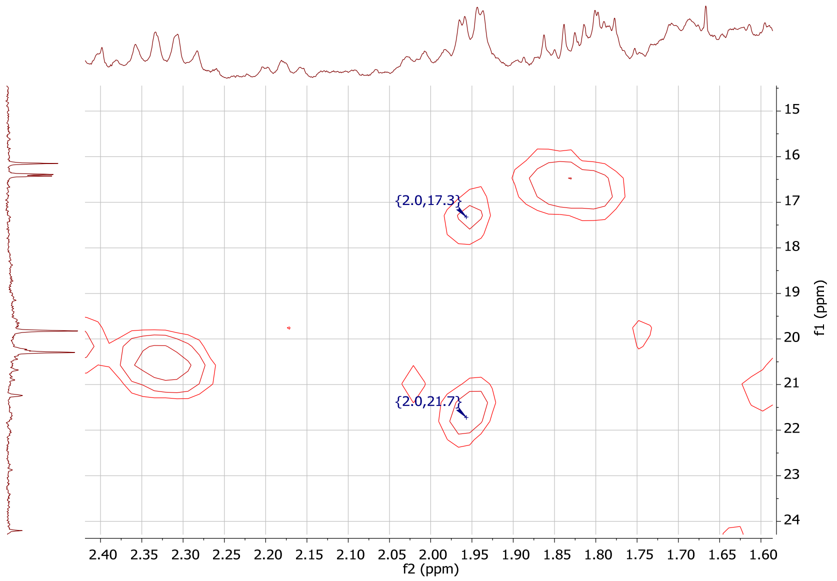

Figure S34. HMBC $\{^1H, ^{13}C\}$ NMR spectrum (300.13, 75.48 MHz, C_6D_6, 298 K) of the mixture of complexes **8** and **4**. Signals corresponding to the $P(CH_3)C{=}CH_2$ are marked.

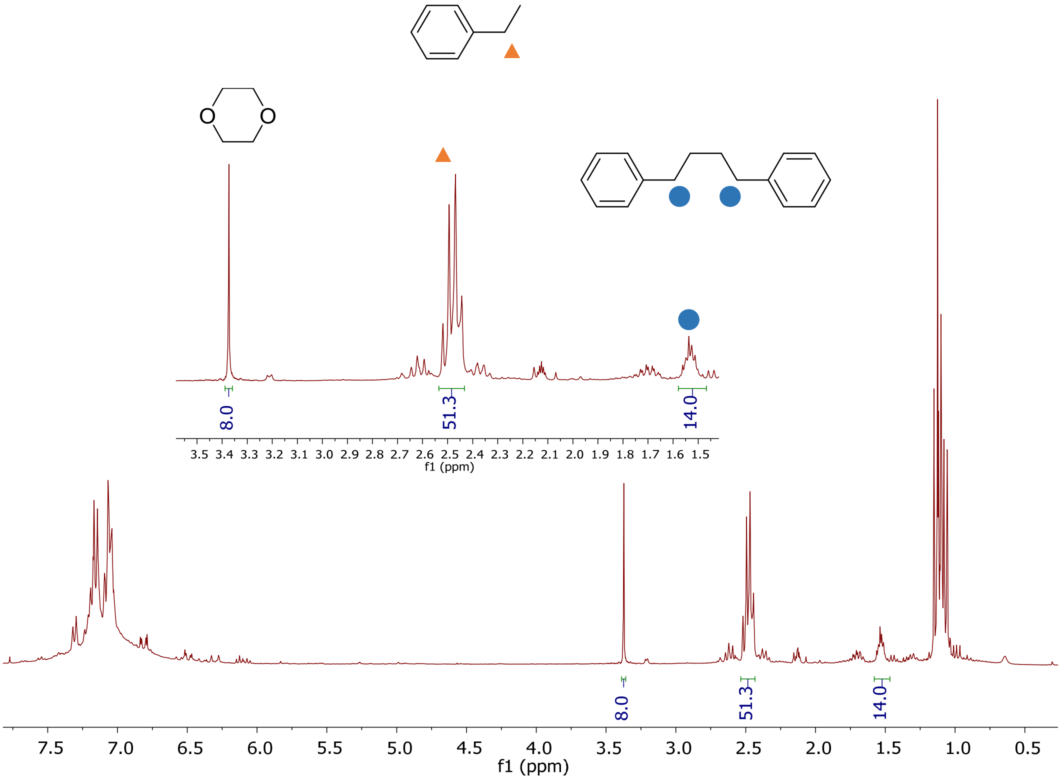

Figure S35. ^{1}H NMR spectrum (300.13 MHz, C_6D_6, 298 K) of the catalytic reaction crude of phenylacetylene. Characteristic resonances used for the calculation of the reaction conversion are marked: two aliphatic CH_2 of the reductive coupling product (blue spot), the aliphatic CH_2 of the alkane (orange triangle).

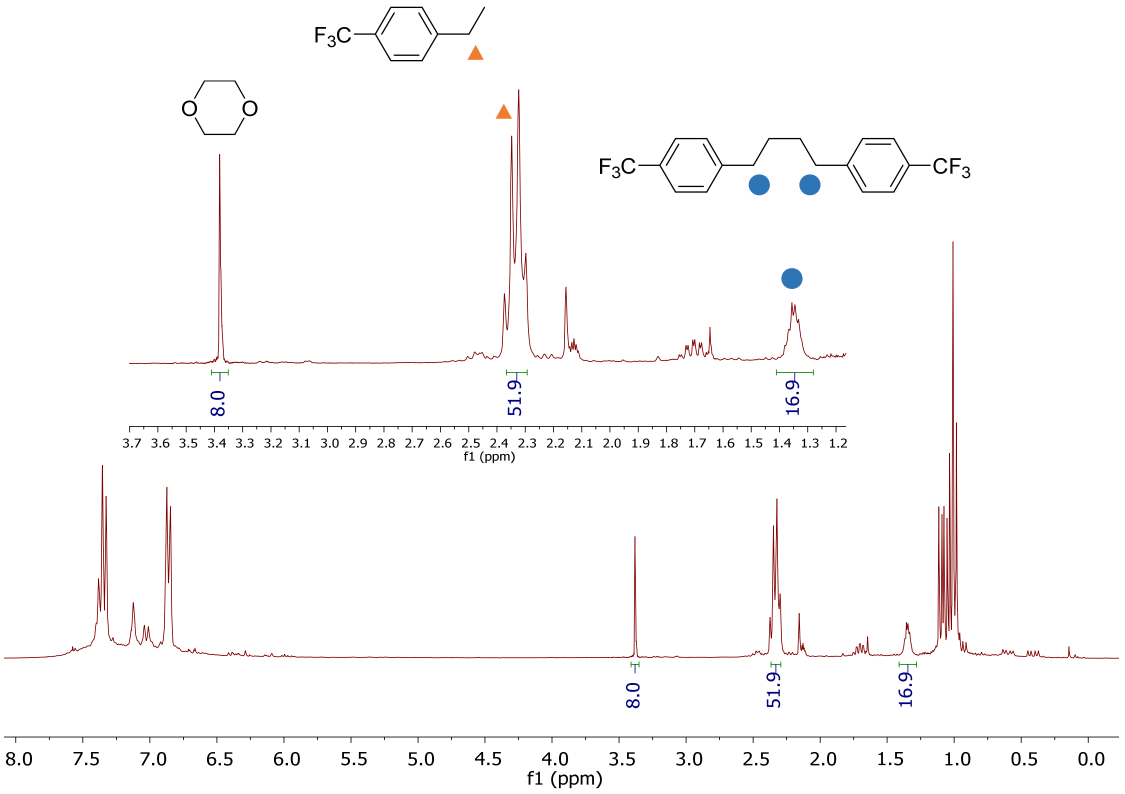

Figure S36. ^{1}H NMR spectrum (300.13 MHz, C_6D_6, 298 K) of the catalytic reaction crude of 4-(trifluoromethyl)phenylacetylene. Characteristic resonances used for the calculation of the reaction conversion are marked: two aliphatic CH_2 of the reductive coupling product (blue spot), the aliphatic CH_2 of the alkane (orange triangle).

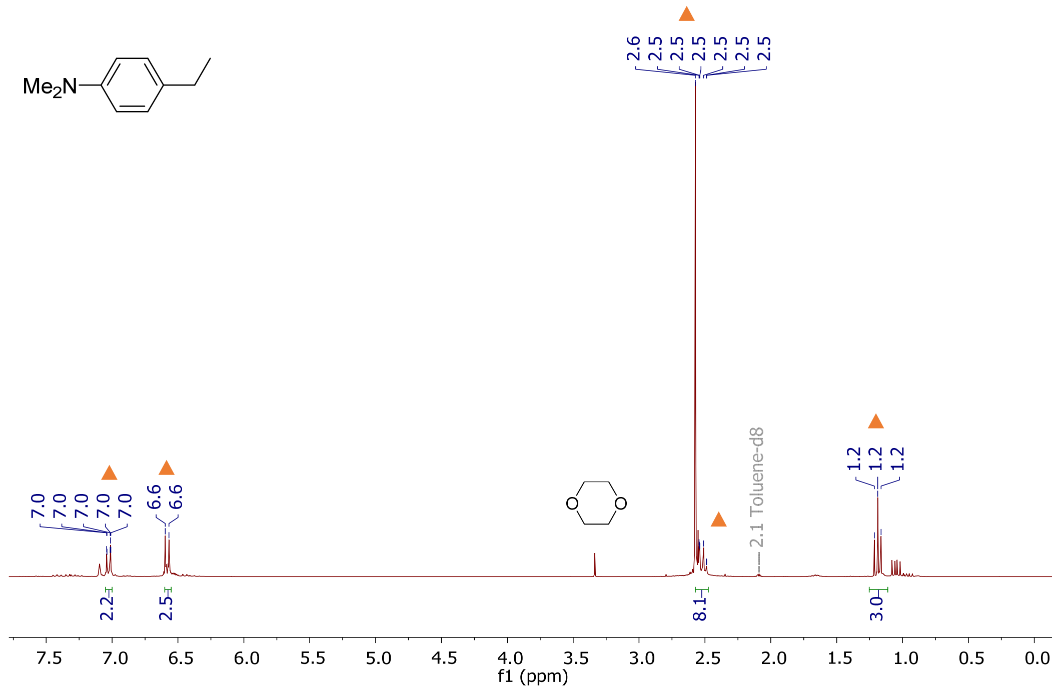

Figure S37. ^{1}H NMR spectrum (300.13 MHz, C_6D_6, 298 K) of the catalytic reaction crude of 4-(dimethylamine)phenylacetylene. Signals corresponding with the hydrogenated product are marked (orange triangles).

References

(1) Aracama, M.; Esteruelas, M. A.; Lahoz, F. J.; López, J. A.; Meyer, U.; Oro, L. A.; Werner, H. Synthesis, Reactivity, Molecular Structure, and Catalytic Activity of the Novel Dichlorodihydridoosmium(IV) Complexes $OsH_2Cl_2(PR_3)_2$ (PR_3 = P-i-Pr_3, PMe-t-Bu_2). *Inorg. Chem.* **1991**, *30*, 288−293.

(2) Blessing, R. H. *Acta Crystallogr.* **1995**, *A51*, 33. SADABS: Area-detector absorption correction; Bruker- AXS, Madison, WI, 1996.

(3) SHELXL-2016/6. Sheldrick, G. M. *Acta Cryst.* **2008**, *A64*, 112-122.

(4) (a) Lee, C.; Yang, W.; Parr, R. G. Development of the Colle-Salvetti correlation energy formula into a functional of the electron density. *Phys. Rev. B* **1988**, *37*, 785– 789. (b) Becke, A. D. Density-functional exchange-energy approximation with correct asymptotic behavior. *J. Chem. Phys*. **1993**, *98*, 5648–5652. (c) Stephens, P. J.; Devlin, F. J.; Chabalowski, C. F.; Frisch, M. J. Ab Initio Calculation of Vibrational Absorption and Circular Dichroism Spectra Using Density Functional Force Fields. *J. Phys. Chem*. **1994**, *98*, 11623–11627.

(5) Grimme, S.; Antony, J.; Ehrlich, S.; Krieg, H. A consistent and accurate ab initio parametrization of density functional dispersion correction (DFT-D) for the 94 elements H-Pu. *J. Chem. Phys*. **2010**, *132*, 154104.

(6) Gaussian 09, Revision D.01, Frisch, M. J.; Trucks, G. W.; Schlegel H. B.; Scuseria, G. E.; Robb, M. A.; Cheeseman, J. R.; Scalmani, G.; Barone, V.; Mennucci, B.; Petersson, G. A.; Nakatsuji, H.; Caricato, M.; Li, X.; Hratchian, H. P.; Izmaylov, A. F.; Bloino, J.; Zheng, G.; Sonnenberg, J. L.; Hada, M.; Ehara, M.; Toyota, K.; Fukuda, R.; Hasegawa, J.; Ishida, M.; Nakajima, T.; Honda, Y.; Kitao, O.; Nakai, H.; Vreven, T.; Montgomery, J. A.; Peralta, Jr., J. E.; Ogliaro, F.; Bearpark, M.; Heyd, J. J.; Brothers, E.; Kudin, K. N.; Staroverov, V. N.; Keith, T.; Kobayashi, R.; Normand, J.; Raghavachari, K.; Rendell, A.; Burant, J. C.; Iyengar, S. S.; Tomasi, J.; Cossi, M.; Rega, N.; S43 Millam, J. M.; Klene, M.; Knox, J. E.; Cross, J. B.; Bakken, V.; Adamo, C.; Jaramillo, J.; Gomperts, R.; Stratmann, R. E.; Yazyev, O.; Austin, A. J.; Cammi, R.; Pomelli, C.; Ochterski, J. W.; Martin, R. L.; Morokuma, K.; Zakrzewski, V. G.; Voth, G. A.; Salvador, P.; Dannenberg, J. J.; Dapprich, S.; Daniels, A. D.; Farkas, O.; Foresman, J. B.; Ortiz, J. V.; Cioslowski, J.; Fox, D. J. Gaussian, Inc., Wallingford CT, 2013.

(7) Andrea, D.; Häußermann, U. M.; Dolg, M.; Stoll, H.; Preuss, H. Energyadjusted ab initio pseudopotentials for the second and third row transition elements. *Theor. Chim. Acta* **1990**, *77*, 123–141.

(8) Ehlers, A. W.; Bohme, M.; Dapprich, S.; Gobbi, A.; Hollwarth, A.; Jonas, V.; Kohler, K. F.; Stegmann, R.; Veldkamp, A.; Frenking, G. A set of f-polarization functions for pseudo-potential basis sets of the transition metals SC-Cu, Y-Ag and La-Au. *Chem. Phys. Lett.* **1993**, *208*, 111–114.

(9) (a) Hehre, W. J.; Ditchfield, R.; Pople, J. A. Self-Consistent Molecular Orbital Methods. XII. Further Extensions of Gaussian-Type Basis Sets for Use in Molecular Orbital Studies of Organic Molecules. *J. Chem. Phys*. **1972**, *56*, 2257–2261. (b) Francl, M. M.; Pietro, W. J.; Hehre, W. J.; Binkley, J. S.; Gordon, M. S.; DeFrees, D. J.; Pople, J. A. Self-consistent molecular orbital methods. XXIII. A polarization-type basis set for second-row elements. *J. Chem. Phys.* **1982**, *77*, 3654–3665.
